# Distributive kind predication

**DOI:** 10.1007/s11050-025-09245-8

**Published:** 2026-03-02

**Authors:** Janek Guerrini

**Affiliations:** Università di Padova, Padua, Italy

**Keywords:** Kind predication, Genericity, Plural predication, Nominals, Bare plurals

## Abstract

This paper makes two contributions to the study of the interpretation of nominals across Germanic and Romance languages. First, it shows that plural kind terms, such as English bare plurals (e.g., *lions*) and Italian definite plurals (e.g., *i leoni*), have definite, non-generic uses in sentences expressing generalizations that were traditionally thought to uniformly involve generic quantification. These non-generic uses explain why the distribution of kind-denoting plurals in sentences expressing generalizations is wider as compared to singular indefinites, which can only appear in generalizations that have a genuinely generic Logical Form (Sects. 3-5). Second, the paper draws on a contrast between English and Italian (and to a minor extent French) plural forms, both bare and definite (Sect. 5). This yields a new approach to the mapping between the form and the interpretation of nominals that combines elements from Chierchia’s (Natural Language Semantics 6:339–405, 1998) and Longobardi’s (Natural Language Semantics 9:335–369, 2001) frameworks. On the approach I present, English bare plurals can alternatively be mapped to kinds or to properties; Italian definite plurals, when not referential, can only be mapped to kinds; and Italian bare plurals only to properties. This explains the behavior of these expressions in a wide number of contexts, most importantly in episodic sentences, which raise puzzles for extant accounts.

## Introduction

Standard theories of genericity take plural generics to have essentially the same logical form as singular indefinite generics, as illustrated in (1)and (2)(Krifka et al. 1995).

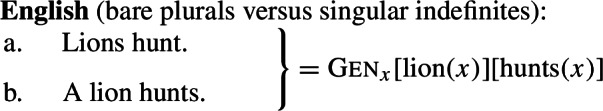

(2)
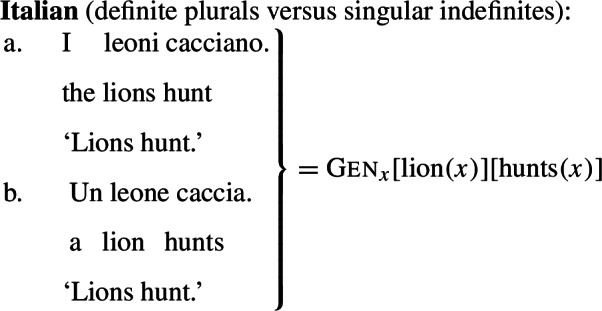

It has long been known that this cannot be the case, as the distribution of generics involving kind-denoting plurals is very different from that of singular indefinites. In this paper, I show that generalizations with kind-denoting plurals do not necessarily involve generic quantification as in (1a) and (2a). Instead, they can involve forms of kind predication mediated by a distributive or cumulative operator, which makes their LFs very close to the LFs of sentences with referential plurals such as ‘the lions’. This explains the difference in distribution between kind-denoting plurals and singular indefinites.^1^

### The difference in distribution between generalizations with kind-denoting plurals and singular indefinites

Kind-denoting plurals and singular indefinites diverge in at least three ways. First, while both singular and plural generics are compatible with law-like generalizations, only plural ones can capture accidentally flavored generalizations (Lawler 1973; Burton-Roberts 1977; Cohen 2001; Greenberg 2002, 2004).^2^ The facts in Italian are parallel to those in English.

(3)
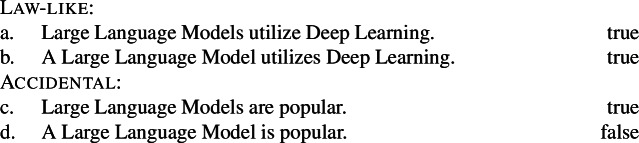

Secondly, plural generics, but not singular indefinite generics, are compatible with cumulative predication (Nickel 2008; Kirkpatrick 2022). The facts in Italian are parallel to those in English.

(4)
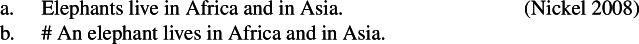

Thirdly, unlike singular indefinites, English bare plurals can be read near-universally in sentences where there is no generic quantification. These are sentences with stage-level predicates (Condoravdi 1994) and with verbs with episodic aspect (Dayal 2013; Chierchia 2022).

(5)

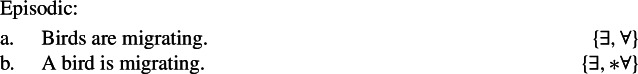

(6)
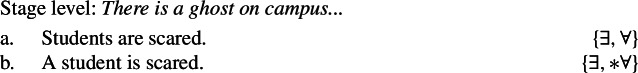

The main reason to think that there is no generic quantification in (5a) or (6a) is to be found in the absence of Quantificational Variability Effects (QVEs) with overt quantificational adverbs (see Berman 1991; Condoravdi 1994). This is because we know that ***Gen*** is a covert quantificational adverb (Lewis 1986; Krifka et al. 1995), and therefore expect it to pattern with its overt cousins. In short, uncontroversial generic sentences pattern as in (7): (7b), constructed by adding ‘rarely’ to (7a), has a reading whose meaning is very close to ‘few birds fly’.

(7)
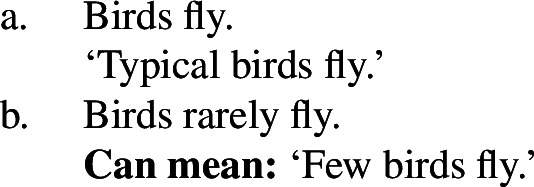

By contrast, if we add ‘rarely’ to (5a), we do not get such a reading (and similarly for (6a)).

(8) . # Birds are rarely migrating.
  **Cannot mean:** ‘Few birds are migrating.’

### The parallelism between referential and kind-denoting plurals

I argue that English bare plurals and Italian definite plurals have these properties simply because they are plurals. More specifically, on one parse of ‘lions hunt’, the sentence is nearly equivalent in meaning to ‘the lions (*of the entire world*) hunt’. Using tools independently motivated in the analysis of referential plurals, i.e., the distributive and cumulative operators, we can derive the contrasts in (3)-(6).

Claims that kind terms can receive interpretations close to referential plurals have appeared before, especially for the first and third puzzles. Krifka et al. (1995) briefly suggest that the contingent flavor of genericity may result from direct kind predication rather than generic quantification. Mari (2010) defends a similar view for Italian definite plurals, focusing on differences in exception tolerance between singular indefinites and plural generics. Dayal (2013) proposes that with non-generic predicates (e.g., episodic or stage-level, as in (5)–(6)), English bare plurals are interpreted as the maximal sum of members of the kind at the evaluation situation. Here, I pursue this idea in full generality. Combined with the proposal that predicates distribute over such sums, it allows us to capture the full range of distributional differences between singular indefinites and kind-denoting plurals across flavors of generalizations (Sect. 3), cumulativity (Sect. 4), and episodic sentences (Sect. 5). Considering episodic sentences will also allow us to revisit the different mapping between form and interpretation in English and Italian nominals (also Sect. 5).

## Assumptions

For concreteness, I will make three formal assumptions:

(9)
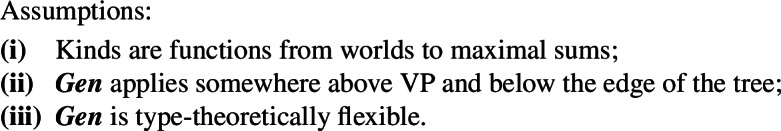

This results in a framework similar (though not identical) to Chierchia’s (1998) formalism concerning kinds and genericity, but nothing in the insight illustrated in the introduction hinges on stipulating a specific architecture.^3^ In fact, only the following two facts are required for a framework to be compatible with the claims made in this paper:

(10)
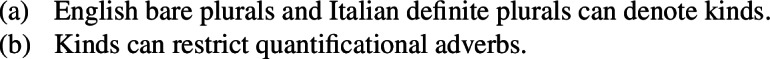

Fact (10a) guarantees that English bare plurals and Italian definite plurals can denote sums of individuals once their world variable has been saturated. This is needed because dist applies to sums. Fact (10b) guarantees that they can restrict ***Gen*** (as ***Gen*** is a silent quantificational adverb; see Krifka et al. 1995, among many others).

Fact (10a) is independently motivated by the acceptability of sentences such as ‘dodos are extinct’ and its Italian equivalent. Fact (10b) is motivated by the acceptability of sentences such as (11)and (12):

(11)

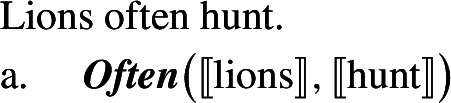

(12)
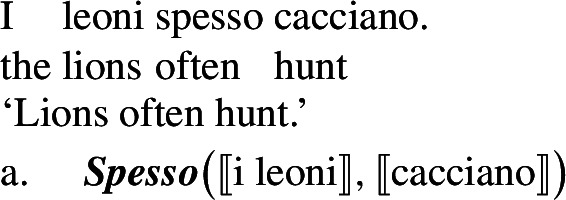

Let us now turn to introducing the three assumptions listed in (9).

**Assumption (i): kind formation**

Nouns in number marking languages like English and Italian start out as properties. An operation of kind formation can turn them into kinds:

(13)
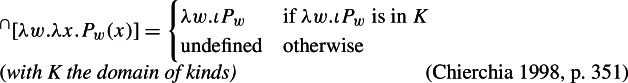

Depending on specific parameters, a language either achieves this via the definite article, as Italian does, or has it apply covertly, as English does (see Chierchia 1998; Dayal 2004b; Cohen 2020).

**Assumption (ii): kinds within generics**

Generic generalizations feature a silent quantificational adverb that has a meaning close to that of a modalized universal quantifier like *generally* (Lewis 1986; Krifka et al. 1995).  The scope of ***Gen*** is its c-command domain, while its restriction is what locally c-commands it (following Chierchia 1995, 1998).

(14)

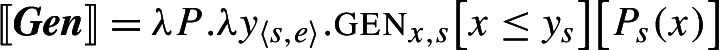

(15)
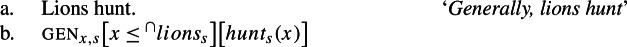

And this generalizes to Italian definite plurals.

I use the modal quantifier gen as a black box, since its interpretation is a very broad and debated issue in itself. See, for instance, the introduction to Mari et al. (2012) for an extensive literature review, and Krifka et al. (1995) and Asher and Morreau (1995) for two concrete proposals. Importantly, I assume that gen uniformly encodes a form of strong law-likeness.

**Assumption (iii)** **: singular indefinites**

The singular indefinite cannot denote a kind, as it does not support kind predication.^4^

(16)
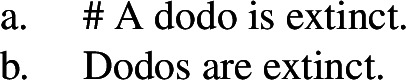

However, it can participate in generic readings.

(17)
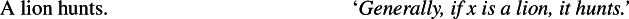

DPs headed by other determiners can also enter the restriction of ***Gen***.^5^

(18) . Two pretenders to the throne hate each other.
  ‘*Generally, if x and y are pretenders to the throne, they hate each other.*’

One could view ***Gen*** as simply unselective, as in Lewis (1986), and thus binding any open variables. In this case, its multiple indexing in LFs would simply be an explicit indication of which variables are being bound. On a different approach, ***Gen*** is selective, and its multiple indexing reflects its compositional behavior (see, for instance, Chierchia 1995). I will not take a stance on these issues here; for compositional clarity, though, I will assume a perspective in which ***Gen*** is selective. In this perspective, one must assume that ***Gen*** is polymorphic, as it can take as an input both kind-denoting plurals and quantificational determiners.

For simplicity, I will here assume that determiners that can enter the restriction of ***Gen*** provide it with a property (see, e.g. van Geenhoven 1998). Almost equivalently, one could assume they denote generalized quantifiers, and that ***Gen*** type-shifts them into a property (as in, e.g., Chierchia 1995). Either way, we expect an alignment between determiners that can enter the restrictor of ***Gen*** and determiners that can receive property-level interpretations, e.g., in copulas, which seems to be correct (see fn. 5).

Plural generics and singular indefinite generics thus wind up having very similar meanings, although the two compositions differ.^6^

(19)

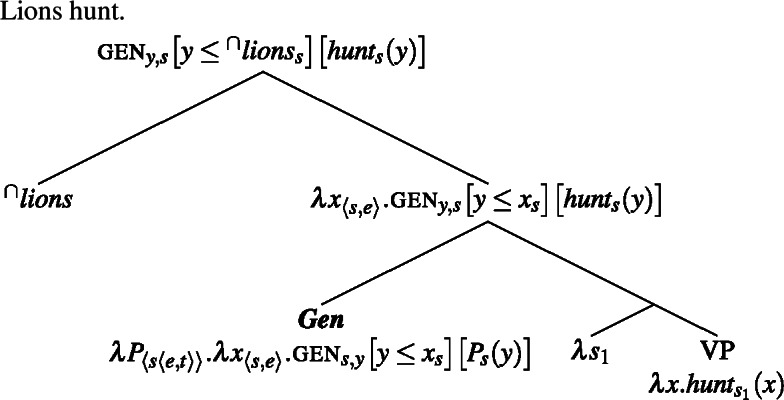

(20)
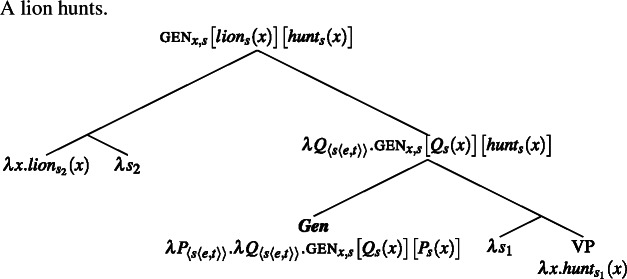

One final caveat concerns the treatment of ***Gen*** as an intensional operator quantifying over worlds (and individuals), following Krifka et al. and Chierchia (1998). Others instead take ***Gen*** to quantify over events (e.g., de Swart 1991) or situations (e.g., Dayal 2004a). This distinction is not crucial here. What matters is that kind-denoting expressions need not appear in the restriction of ***Gen***, since predicates can distribute over them directly. This is compatible with all of the above frameworks. Given the diversity of views on genericity, the formal choices made here are for concreteness, not a theoretical commitment.

## Accidentally flavored generalizations with kind-denoting plurals

### Flavors of genericity

Consider (21) and (22).^7^

(21)

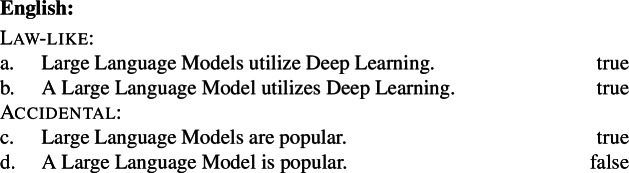

(22)
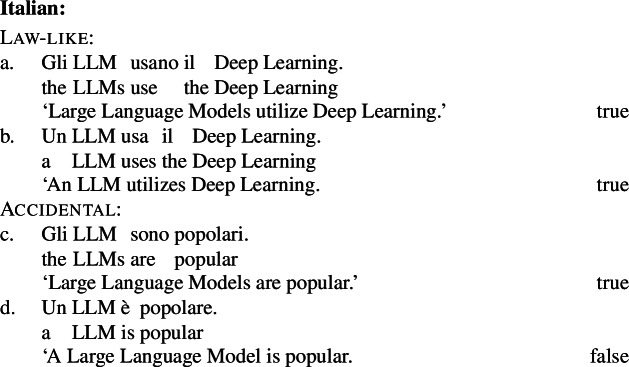

The common pattern is summarized in Table 1.

**Table 1:** Summary of the behavior of kind-denoting plurals and singular indefinites concerning the flavors of generalizations in which they appear

	Law-like flavor	Accidental flavor
Kind-denoting plural	✓	✓
Singular Indefinite	✓	*

There is no consensus analysis of this contrast. Krifka et al. (1995) first raised the possibility that accidental readings of kind-denoting plurals may be felicitous because they involve direct kind predication, as in (23), and not generic quantification, as in (24) (see Carlson 1977 too; see also Liebesman 2011 for a theory viewing all generic sentences as non-quantificational, i.e., as instances of direct kind predication).

(23)

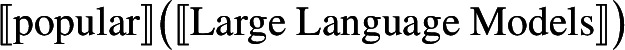

(24)
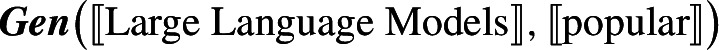

A number of criticisms of this idea were raised in the literature. Cohen (2001) observed that clear direct kind predication with bare plurals resists modification by Q-adverbs, as shown in (25a), unlike characterizing sentences, as in (25b).^8^ According to Cohen, since ***Gen*** is a silent Q-adverb, this suggested that it is absent in kind predication, but present in accidental generalizations.

(25)
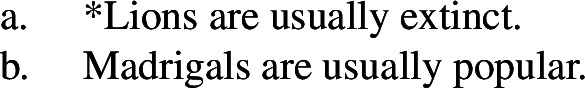

Cohen also observed that sentences like (26)involve scope ambiguities, which are hard to account if we assume Krifka et al.’s proposed non-quantificational LF.

(26)
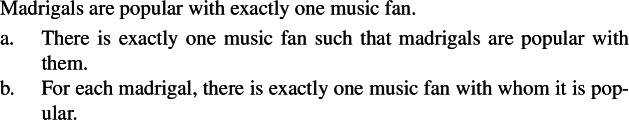

Others have argued against Krifka et al.’s idea by invoking the behavior of bare plurals with respect to binding: (27a) does not mean that the cat kind likes the cat kind, as in (27b) (Chierchia 1998, a.o.).

(27)
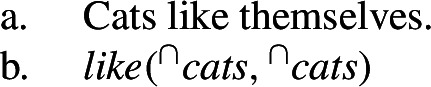

These observations are important, as they show that Krifka et al.’s idea cannot be implemented as is: in general, fully non-quantificational theories of generalizations with kind-denoting plurals are bound to make wrong predictions.^9^ I propose that what we need is *distributive* kind predication, as we will see in the next section. In Sect. 3.6, I will detail how this counters the criticisms above quite naturally.

Following the observations in (25)–(27), two main families of views emerged. Cohen (2001), a proponent of an “ambiguity” theory, argues, partly based on (25)–(27a), that bare plural generics are ambiguous between a “rule” and a probabilistic reading, while singular indefinites express only rules (see also Krifka 2003; Mari et al. 2012). In contrast, Greenberg (2002, 2004, 2007) defends a “one meaning” view: both forms involve Gen, but differ in the accessibility relation introduced by gen, allowing bare plurals to support accidental generalizations. The view I develop is also an “ambiguity” theory, though distinct from Cohen’s and closer in spirit to Krifka et al.’s original proposal, in a way that addresses the criticisms above.

### Analysis

I propose that sentences containing kind-denoting plurals (English bare plurals and Italian definite plurals) are structurally ambiguous between two forms:

(i) one that is parallel to the LF of singular indefinite generics, giving rise to the law-like reading; see (28a).
(ii) one that is parallel to distributive predication with referential plurals, giving rise to the accidental reading; see (28b).

(28)
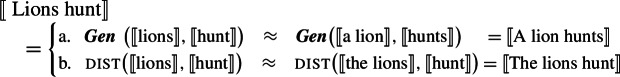

Specifically, in (28a) the world variable of the kind remains abstracted over, and the kind enters the restriction of the generic quantifier. Let us call this the Bona Fide Generic reading. This is the structure familiar from (19). In (28b), the kind is interpreted at the evaluation world $s_{0}$ and the predicate denoted by the verb is distributed over the atoms that are parts of the kind at that world. Importantly, there is thus no generic quantification over members of the kind. Let us call this the Distributive Kind Predication parse. See structure in (30).

(29)

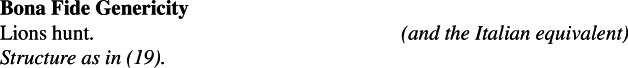

(30)
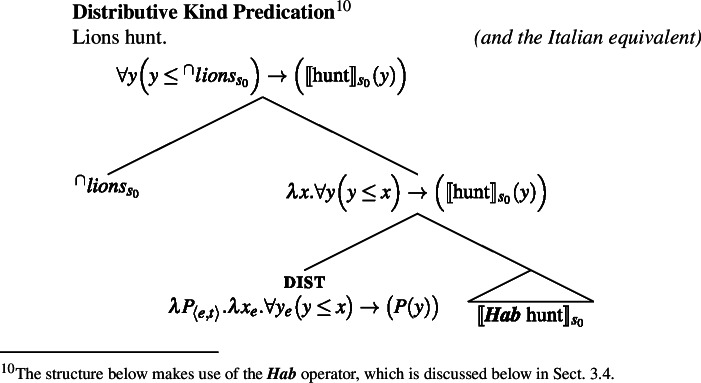

Dist applies to pluralities.^11^^,^^12^ The point made here is simply that this includes kinds with a saturated world variable.^13^ Whether the kind denoted by ‘lions’ is in the restriction of ***Gen*** (and thus has its world variable bound by gen), or has its world variable saturated by the evaluation world determines whether we have a modal generalization or an accidental one: (29)tells us something about the nature of lions; (30)tells us something about the properties of actual lions. This insight does not depend on how we specify the low part of the tree, which is why here it is left underspecified; it only depends on whether or not ‘lions’ is in the restriction of ***Gen***.

At this point, we want to explain why singular indefinite generics cannot receive accidental readings. The singular indefinite cannot denote a kind (nor a sum), and therefore does not support the application of dist. Instead, it can either enter the restriction of the generic quantifier, or acquire existential force via an existentially closed choice function, as is standard (see, e.g., Reinhart 1997; Winter 1997).^14^ The existential reading is very marginal, but generated by the grammar, as it can, e.g., be made more salient with appropriate restrictions (‘A lion I know hunts’).

(31)
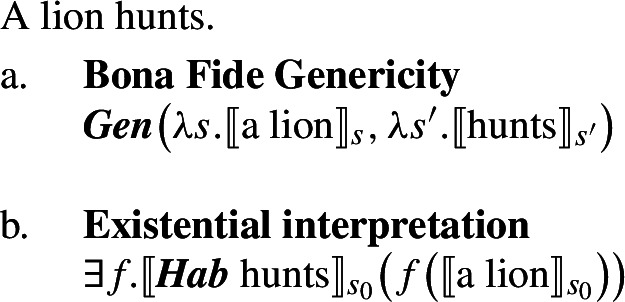

These facts solve the puzzle. The Bona Fide Generic reading is a *modal generalization*, and thus gives us a law-like reading. The distributive predication reading tells us something about the habits of *actual* lions. This is summarized in Table 2.^15^

**Table 2:** Summary of the LFs hypothesized for generalizations with kind-denoting plurals and singular indefinites and the flavors they produce

	Law-like fl./Bona-fide gen.	Accidental fl./ Dist. k. pred.
Kind-denoting Plural	✓	✓
Singular Indefinite	✓	*

### Homogeneity and its removal

As is standard, dist is weaker than a universal quantification such as the one expressed by *each*. When combining with definites, it can be seen as the source of two much discussed phenomena, known as homogeneity and non-maximality. Homogeneity refers to polarity reversals like the one exemplified in (32), where ‘the kids’ behaves near-universally in a positive sentence, but near-existentially in a negative sentence (Schwarzschild 1996; Löbner 2000; Spector 2013; Križ 2015; Feinmann 2020; Križ and Spector 2021; Bar-Lev 2021, among many others).^16^

(32)
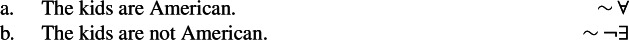

“Non-maximality” refers to exception tolerance: (32a) can be used, in some contexts, even if one or two kids are not American; and (32b) if one or two are (see Lasersohn 1999, a.o.). Homogeneity and non-maximality tend to pattern together (see Malamud 2012; Križ 2015; Križ and Spector 2021, a.o., and much subsequent work). This is clear when we compare (32)to minimally contrasting sentences with ‘all’, where both effects disappear. ‘All’ removes homogeneity (Löbner 2000): negating (32a) yields something like a negated existential, while negating (33a) gives a plain negated universal:

(33)
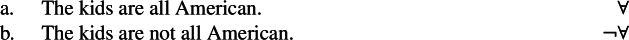

Second, as has been known since Brisson (1998, 2003), *all* also removes non-maximality: (33a) cannot be used if one or two kids are American, unlike (32a) (and similarly for (33b) vs. (32b)) (see also Lasersohn 1999 on “slack regulation”).

The same pattern, concerning both homogeneity and non-maximality, holds for generalizations involving bare plurals (see Löbner 2000).

(34)

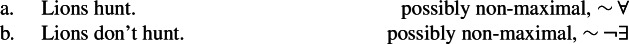

(35)
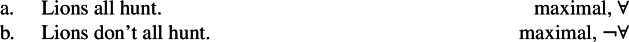

Employing dist ensures that the Distributive Kind Predication LF (see (30)) behaves non-maximally and homogeneously like definites, and that its homogeneity can be removed via ‘all’. Whatever explains homogeneity and non-maximality with definite plurals can be extended to Distributive Kind Predication with bare plurals.

It is well-known that generic sentences, too, are in general homogeneous and non-maximal. While their exception tolerance was among the first facts observed concerning their behavior, dating back at least to Lawler (1973), the fact that they undergo polarity reversals analogous to definite plurals was first noticed by Löbner (2000) and von Fintel (1997).^17^

(36)
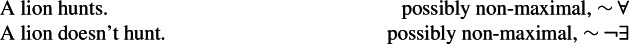

Concerning homogeneity removal, Križ (2015) noticed that generalizations involving bare plurals can have their homogeneity removed both via ‘all’ (see (34)) and via ‘always’, as in (37). Once again, this pattern extends to Italian kind-denoting (definite) plurals.

(37)
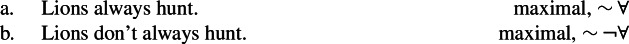

By contrast, the homogeneity of singular indefinites can only be removed via ‘always’.

(38)
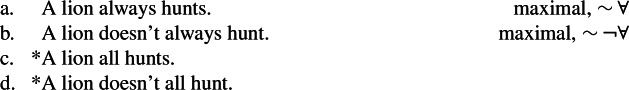

This pattern of homogeneity removal falls out from the present view. Specifically, we can think of ‘all’ as removing homogeneity over individuals, and thus as a non-homogeneous counterpart of dist, as is standard. Additionally, ‘always’ can be seen as removing homogeneity over worlds (or times, or situations), and thus as a non-homogeneous counterpart of ***Gen***, since both are quantificational adverbs. Then, we can articulate the similarities and differences between (i) sentences involving referential plurals, (ii) generalizations involving singular indefinites, and (iii) generalizations involving kind-denoting plurals.^18^

(i) sentences with referential plurals (definite plurals in English and Italian), whose LF unambiguously involves dist (when combining with predicates of individuals), can have their homogeneity removed by ‘all’, but not by ‘always’;
(ii) generalizations involving singular indefinites, whose LF involves ***Gen*** but not dist, can have it removed via ‘always’, but not via ‘all’;
(iii) generalizations with kind-denoting plurals (bare plurals in English, definite plurals in Italian) are structurally ambiguous between two forms:
  an LF involving dist, whose homogeneity can be removed via ‘all’;
  an LF involving ***Gen***, whose homogeneity can be removed via ‘always’.

This is summarized in Table 3.

**Table 3:** Summary of possible homogeneity removers for sentences with definite plurals, generalizations with singular indefinites, and generalizations with kind-denoting plurals

	homogeneous LF(s)	Corresponding homogeneity remover(s)
Sentences with referential definite plurals	dist	‘all’
Generalizations with singular indefinites	***Gen***	‘always’
Generalizations with kind-denoting plurals	dist; ***Gen***	‘all’; ‘always’

### Genericity and habituality

Let us now consider the two structures we have posited in Sect. 3.2 for ‘lions hunt’, and comment on why we have 〚hunt〛 in the low part of the tree of the Bona Fide Genericity LF in (29), but 〚***Hab*** hunt〛 in the low part of the tree in the Distributive Kind Predication LF in (30). I include this discussion for theoretical completeness, to show that the low part of the tree does not affect any of the paper’s main claims. Readers who are mainly interested in the main line of argument may therefore skip ahead to the next section.

On a prominent view of genericity, ***Gen*** is brought about by lexical aspect: this is what explains that aspect itself determines whether an indefinite is more saliently interpreted generically or existentially in a given context (Krifka et al. 1995).

(39)
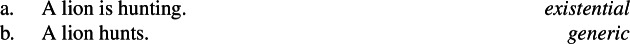

Because no ***Gen*** appears in the Distributive Kind Predication LF in (30), I signal the presence of habitual aspect by writing 〚***Hab*** hunt〛. In fact, on Chierchia’s (1995, 1998) theory, ***Hab*** would be itself ***Gen***, as Chierchia views instances of habitual aspect such as ‘John smokes’ as quantifying generically over situations involving John.^19^

(40)
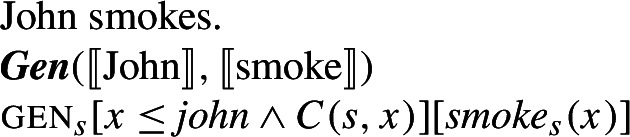

This would give us a view in which what distinguishes the Distributive Kind Predication LF from the Bona Fide Generic parse is simply the optional insertion of dist above ***Gen***, while the low part of the tree is common.

(41)

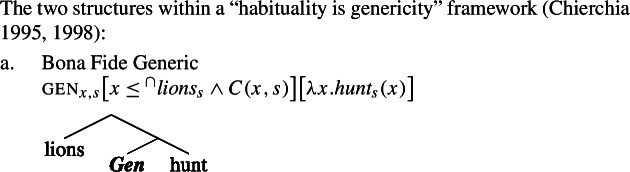

(42)
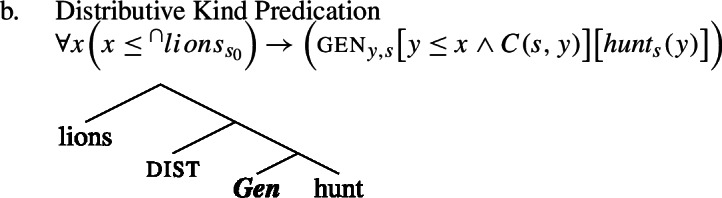

Against this view, others view genericity as entirely distinct from lexical aspect. Thus, on, e.g., Dobrovie-Sorin’s (2001) approach, the habitual operator ***Hab*** would be distinct from ***Gen***, and in fact appear below ***Gen*** in (29). The habitual operator is seen as the single operator contributed by lexical aspect. For another view that takes habitual aspect to be different from Hab, see Boneh and Doron (2012). For ***Hab*** itself, see Schoorlemmer (1995), Dobrovie-Sorin (2001), Scheiner (2002), Spector (2003), van Geenhoven (2004), and Rimell (2005), all cited in Boneh and Doron (2012).

In this framework, we would have ***Gen*** and dist be *alternatively* inserted above the obligatory ***Hab***.

(43)
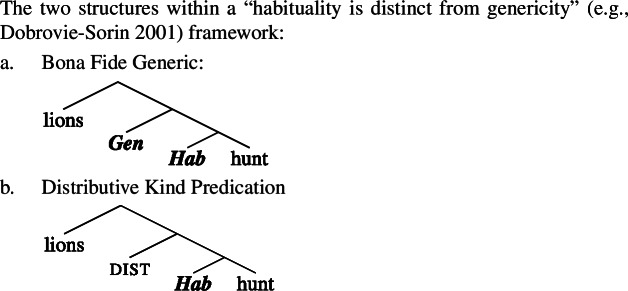

The fundamental insight of the present analysis is that kind-denoting plurals are interpreted inside the restriction of ***Gen*** in law-like generalizations and outside ***Gen*** in accidental ones. This does not hinge on which of these theoretical options one chooses concerning the relationship between habituality and genericity. For ease of exposition, in the paper I will keep simplifying low parts of the tree as in Sect. 3.2 above.

### Law-likeness and the Italian subjunctive

The crucial feature of the present account is that the subject DP is interpreted inside the restrictor of ***Gen*** only in the Bona Fide Generic parse. We therefore generate an additional prediction. We expect that if anything in the subject DP is subject to licensing by ***Gen***, it should only be licensed in the Bona Fide Generic parse.

This is the case for the Italian subjunctive. The subjunctive is licensed in Romance in broadly intensional environments; the parameter that changes across Romance is which environments specifically license it. In Italian, the restrictor of the generic quantifier is among these licensing environments (Farkas 1981; Panzeri 2006).

(44)
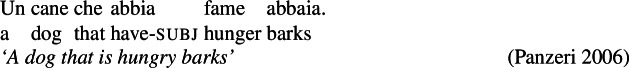

This prediction is correct: when the subject DP is modified by a relative in the indicative mood, the sentence is compatible with both readings. When, instead, it is modified by a relative in the subjunctive, the sentence is only compatible with a law-like reading.^20^

(45)
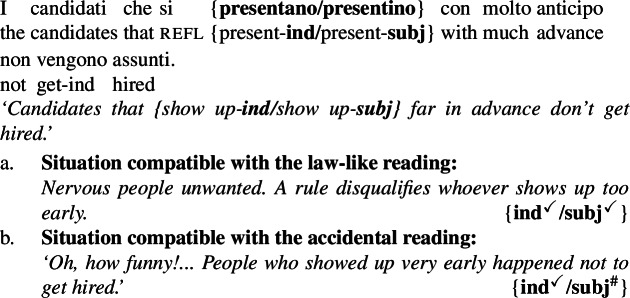

The reasoning is as follows: we have hypothesized that accidental readings stem from an LF involving distributive predication over actual members of the kind. These are LFs in which the kind-denoting plural is not in the restictor of ***Gen***, and thus don’t license the subjunctive. This leaves the Bona Fide Genericity LF as the only one available; as a result, the sentence only has a law-like reading.

- (44) Distributive Kind Predication parse, therefore:
  Subjunctive outside the restriction of ***Gen***, therefore:
  subjunctive not licensed (indicative always licensed)
- (44) Bona Fide Generic parse, therefore:
  Subjunctive inside the restriction of ***Gen***, therefore:
  subjunctive licensed (indicative always licensed)

### Countering past criticisms of the idea of kind predication in accidental generalizations

Now that we have articulated and motivated the idea of a structural ambiguity, let us go back to Cohen and Chierchia’s criticisms of Krifka et al.’s original idea of direct kind predication mentioned in Sect. 3.1 ((25)-(27)) and summarized in (46).

(46)
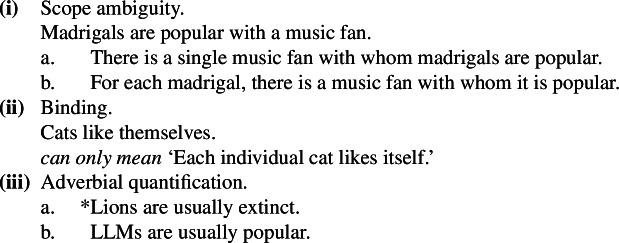

Mediating kind predication via the distributive operator explains these facts very naturally. Concerning the fact that bare plurals are scopally ambiguous with respect to other indefinites, we know independently that indefinites can take scope either below or above dist.

(47)
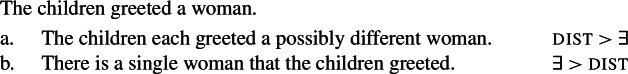

The scope ambiguity of sentences like (26) is thus entirely expected.

(48)

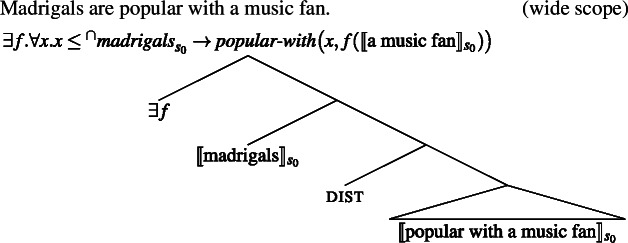

(49)

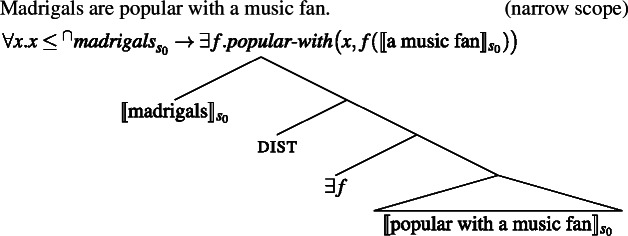

**(ii)** Concerning the fact that reflexives seem to be bound “individually” (and not kind-wise), because we resort to dist, we correctly predict that ‘cats like themselves’ can mean, in its Distributive Kind Predication parse, that every subpart *x* of [the cat kind interpreted at the evaluation world] likes *x*.

**(iii)** Lastly, let me discuss the fact that direct kind predication with clear kind predicates like ‘extinct’ resists adverbial quantification, while predicates like ‘popular’ don’t. Cohen claimed that Krifka et al.’s idea of kind predication was incorrect because bare plurals can be bound by overt Q-adverbs in sentences with “accidental” predicates, but not in sentences with clear kind-level predicates. In the present theory, this is explained by the fact that overt Q-adverbs can replace ***Gen*** in a parse parallel to the Bona Fide Generic parse.

(50)
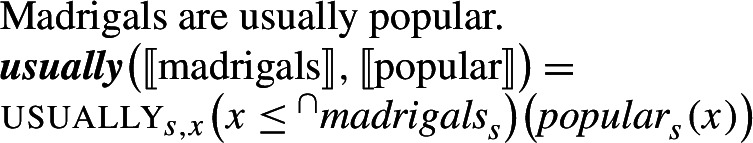

Cohen (2001, p. 189) argues from **(i)**–**(iii)** for a complex mechanism: kind predication (e.g., $\textit{popular}_{s_{0}}({^{\cap}madrigals})$) is ruled out, since only individuals can be popular, and an LF with ***Gen*** is pragmatically accommodated to bind instances. But, if predicates distribute over pluralities as in my account, kind predication need not be excluded in the first place, and Cohen’s additional accommodation step becomes unnecessary.

### Further data: Greenberg (2002, 2004, 2007)

In her work, Greenberg presented remarkable data points pertaining to (non-)accidentally flavored generalizations, further teasing apart kind-denoting plurals from singular indefinites.

(51)

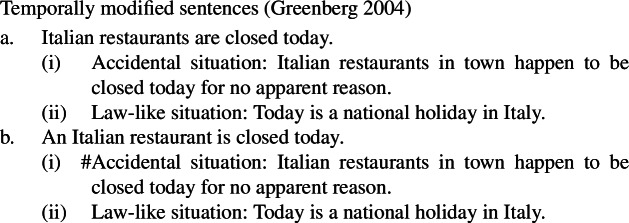

(52)
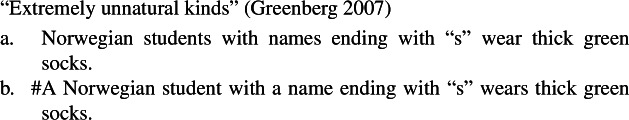

This fits the present framework. LFs with the subject DP inside ***Gen*** require a law-like link between membership in the subject’s extension and having the VP property. But kind-denoting plurals also allow an LF with the subject outside ***Gen***, so Distributive Kind Predication makes (50a) (and (51a)) true even in the absence of such a law-like link.^21^

(53)
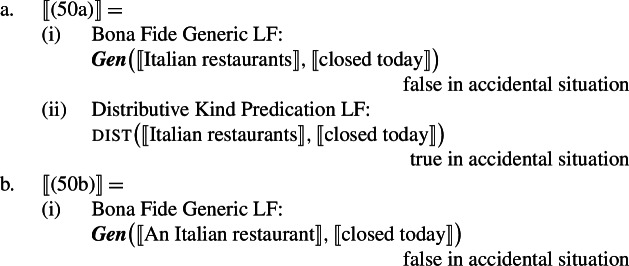

Similarly, in (52), there is no law-like link between being a Norwegian student with a name ending in “s” and wearing thick green socks, so a generic LF is false; but the Distributive Kind Predication LF of (53a) does not require such a link, whence a true construal. Yet if context supplies such a link, (52b) is fine:

(54)
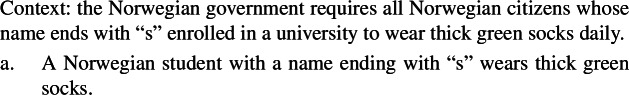

In Greenberg’s framework, ***Gen*** is present in both, but bare plurals need no actual-world association, while singular indefinites rely on norms or stereotypes.

(55)
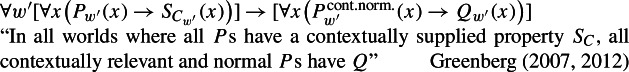

Greenberg’s framework has clarified many differences between singular indefinite and bare plural generics, but it still fails to capture some facts that the present account explains, unless one adds further stipulations.

In particular, in my account accidental readings via Distributive Kind Predication arise compositionally from independently motivated assumptions: (a) bare plurals denote kinds (Carlson 1977), and kinds denote plural individuals (Chierchia 1998); (b) the distributive operator may apply whenever a predicate of individuals targets a plural individual (Schwarzschild 1996). In Greenberg’s account, contrasts in accessibility must be stipulated.

Additionally, as shown in Sect. 5, bare plurals can take near-universal readings in episodic sentences, while singular indefinites are only existential ((56a), (57)). Since episodic bare plurals lack QVEs with overt Q-adverbs, and ***Gen*** is itself a covert Q-adverb, these sentences plausibly lack generic quantification.

(56)

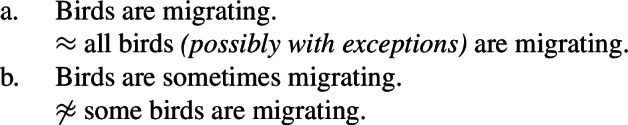

(57)
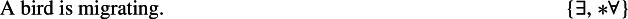

Thus, Greenberg’s account cannot explain the distribution of bare plurals and singular indefinites in such sentences, which is nevertheless parallel to what happens with (non-)accidental generalizations.

## Cumulativity with kind-denoting plurals

### Analysis: cumulative kind predication

Let us now turn to sentences like (58)and (59):

(58)

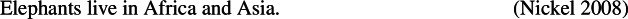

(59)
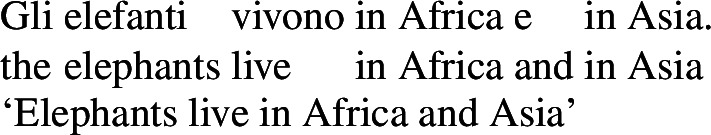

It has been known since the work of Scha (1981) that sentences containing more than one term denoting a sum can give rise to weak truth conditions:

(60) . The girls greeted the boys.
  **Can mean:** Each girl greeted some boy, and each boy was greeted by some girl.

Beck and Sauerland (2000) showed that to account for at least some of such readings, a cumulative operator ∗∗ is needed.

(61)
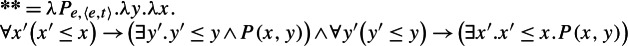

The approach put forward in the previous section can be quite naturally extended to account for cumulative readings of kind-denoting plurals: just like (59) can be captured with the structure in (62), (57) can be captured with the structure in (63).

(62)

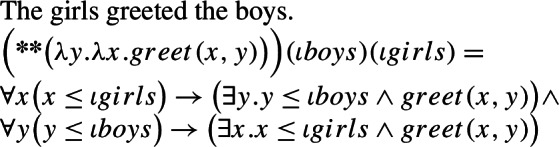

(63)
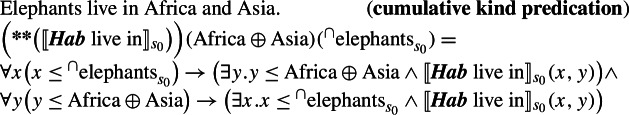

If we specify the low part of the tree, i.e., if we specify 〚(***Hab***) live in〛 as a generic/habitual predicate in a way parallel to what we have sketched in Sect. 3.4, *elephants* and *Africa and Asia* should QR above ***Gen***.^22^

(64) .

At this point, we know that for kind predication, along a distributive LF, there is a cumulative LF available. But what about Bona Fide Generic LFs?

The line I will pursue here involves thinking that ***Gen*** does *not* encode cumulativity in its semantics, a hypothesis that I now turn to discussing in detail.

### No cumulativity with Bona fide generic LFs and overt Q-adverbs

Alongside cumulativity that is independent of ***Gen***, as in the LF I presented in (62), it has been long known that there can be cumulativity *below* ***Gen*** (Corblin 1987; Dobrovie-Sorin and Mari 2008). This is witnessed by sentences as (65), which can have the reading paraphrased in (65a).

(65)
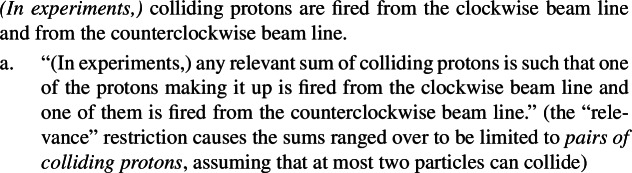

The LF of such sentences can be correctly predicted by simply assuming that ***Gen*** here ranges over sums and that the cumulative operator occurs in the scope of ***Gen*** (that ***Gen*** and other Q-adverbs can range on sums is generally accepted; Dobrovie-Sorin and Mari 2008).^23^

(66)
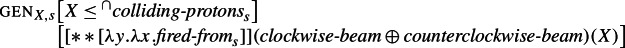

We can also have cumulativity *below* ***Gen*** when the restriction of ***Gen*** is provided by an indefinite numeral (so, say, a property) as in (67)(see Corblin 1987; Dobrovie-Sorin and Mari 2008).

(67)

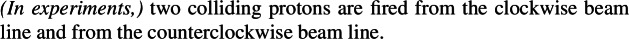

(68)
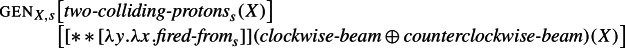

Importantly, the cumulative reading in (65) is much stronger than the salient reading of “elephant” sentences like (57): (68)asserts that any pair of colliding protons comes from opposing beams. (57), by contrast, merely states a global elephant distribution, and not that every elephant sample includes one in Africa and one in Asia.

As a result, we know for sure that there can be cumulation either (i) above habitual marking, as in (62), or (ii) below ***Gen***^24^ as in (64), but neither of these amounts to an LF in which the meaning of ***Gen*** itself gives rise to cumulativity. Looking at (i) and (ii) we see that, if we extend Beck and Sauerland’s approach, the source of cumulativity is simply the cumulative operator **.

We have two ways of checking whether in addition to this, there is a Bona Fide Generic *cumulative* LF. The first consists in checking whether we can get cumulative readings of overt Q-adverbs—since it is generally accepted that ***Gen*** is a covert Q-adverb. While the judgments are subtle, I think they show that Q-adverbs themselves are not cumulative. To see this, consider (69).^25^^,^^26^

(69)
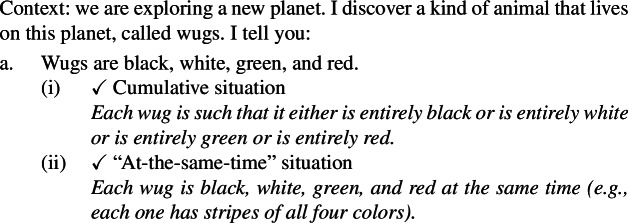

Of course, the cumulative interpretation is, while entirely possible, the less salient one in (68a)—similar facts hold of cumulative predication with referential plurals. What is important is that a cumulative interpretation is completely ruled out with Q-adverbs, as in (70).

(70)
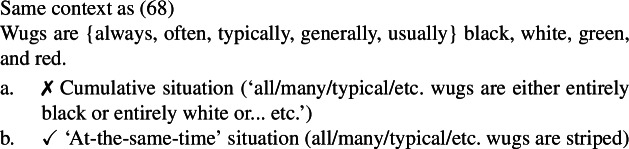

This data suggests that (68a) arises through cumulative kind predication, and not via ***Gen***, unless independent evidence is adduced that motivates that ***Gen*** would pattern unlike its overt cousins (‘always’, ‘often’, etc.) with respect to cumulativity.

A second way we have to check whether cumulative LFs with ***Gen*** obtain is to force a kind-denoting plural to be in the restriction of ***Gen***. We can use the Italian subjunctive for this, as we have seen in Sect. 3.5.

(71)

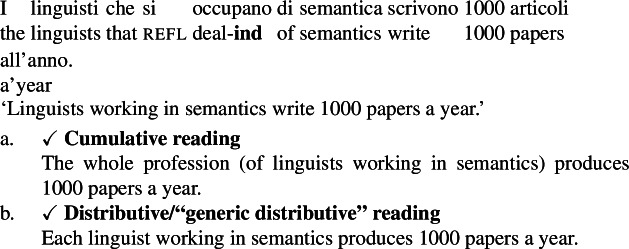

(72)
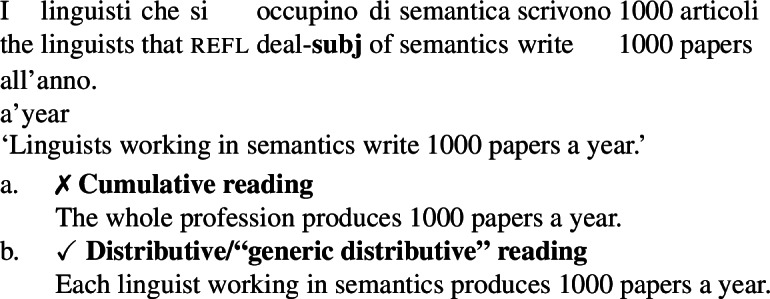

Once again, although the data is subtle, it seems to rule out an LF in which ***Gen*** gives rise to cumulativity independently of ∗∗. Indeed, the cumulative interpretation of (71a) is naturally captured by a cumulative kind predication LF such as (73). (73), in its cumulative reading, tells us that every actual semanticist has written at least one of the 1000 papers, and each one of the 1000 papers was written by at least one semanticist.

(73)
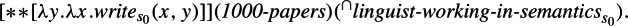

Instead, the Italian subjunctive, as we have seen in Sect. 3.5, forces an LF in which the subject DP is in the restriction of ***Gen***. In fact, as shown by the unavailability of (72a), (71) does not support a cumulative interpretation. This suggests that reading (70a) of (70) can in fact *only* come about from a cumulative kind interpretation LF such as (73).

To sum up, in this section we have investigated which one of the LFs in (74a,b,c) brings about cumulative interpretations.

(74)
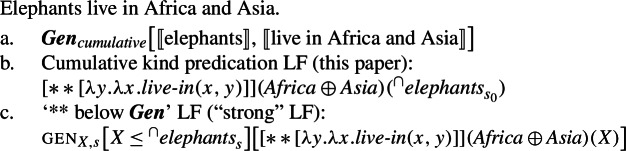

As to (74c), it yields a false construal of (74), since it states that any sample of elephants includes some from Africa and some from Asia. Hence it cannot underlie the salient true reading. LFs with ** below ***Gen*** instead account for strong generalizations such as those in the “firing protons” case, which assert that any sample containing two colliding protons has one fired from the clockwise beam and one from the counterclockwise beam.

As to (74a), the data reviewed here suggest that this LF likewise fails: the semantics of ***Gen*** and other Q-adverbs does not deliver cumulativity. Overt Q-adverbs remove cumulative readings ((68)), even those close in meaning to ***Gen***. Were they to diverge, the absence of cumulativity with Italian subjunctive-modified DPs would be unaccounted for (71).

Thus, the cumulative kind predication LF (74b) remains the only option for the salient construal of ‘elephants live in Africa and in Asia’ and alike sentences.

### Comparison to previous accounts

#### Nickel (2008)

There are two accounts of cumulative readings of sentences with bare plural subjects. Nickel (2008) proposes, in a nutshell, that cumulative readings arise because the generic operator has existential force over the “ways of being normal” of a category.

(75)
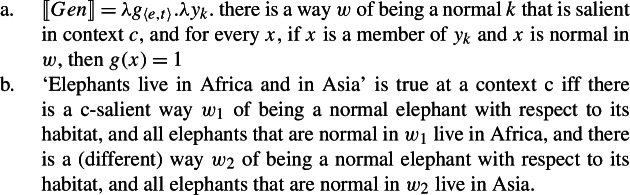

This hinges on Nickel’s assumption that ‘Elephants live in Africa and Asia’ is an ellipsis of the counterpart with sentential coordination (‘Elephants live in Africa and elephants live in Asia’), hence two instances of generic quantification. Kirkpatrick (2022, p. 390) argues, however, that Africa and Asia is clearly a constituent, thus does not admit of phonologically deleted material, and cannot be split as Nickel’s analysis requires.

A reviewer notes that Kirkpatrick’s criticism does not take into account the possibility of gapping (see, e.g., Johnson 2006 for an overview). However, there are more general reasons to think that the sentences under review are not a conjunction of propositions in the relevant readings. There is an exhaustive reading of (76)on which it is false—since Africa is not the only habitat of elephants.

(76)
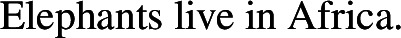

This is witnessed by the felicity of the following responses to (76), confirmed by three native speakers of English:

(77)

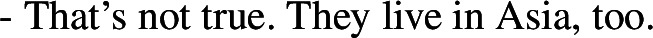

(78)
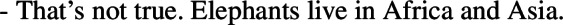

A conjunction reduction structure ruling out the necessity of cumulativity would involve a conjunction of propositions. But this could not be a conjunction of propositions that have an exhaustive reading like the one of (75) that (77)and (78)respond to—the falsity of one conjunct would make the conjunction of propositions false. This shows that there must be an LF on which the single predicate ‘living in Africa and Asia’ is cumulatively (and exhaustively) predicated of elephants.

As a reviewer notes, one might worry that the relevant reading reduces to a conjunction of two non-exhaustive propositions (‘some elephants live in Africa, some in Asia’). But this cannot explain why the cumulative reading can itself be interpreted exhaustively:

(79)
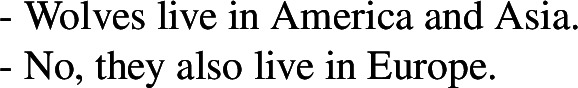

The correction in (79)is felicitous only if the original is taken as false for omitting Europe. A non-exhaustive conjunction (‘some in America, some in Asia’) would remain true here, and thus not invite correction. The cumulative reading itself supports an exhaustive construal, which cannot be reduced to a mere conjunction of non-exhaustive propositions.

#### Kirkpatrick (2022)

Kirkpatrick (2022) proposes a complex situation-based semantics of the generic operator where ***Gen*** operates over properties of situations. The interaction between this entry for ***Gen*** and pluralities generates cumulative readings.

(80)
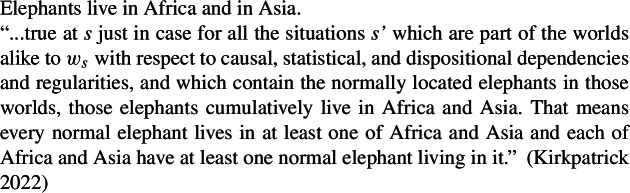

There are two aspects on which some reservations can be expressed regarding Kirkpatrick’s position.

First, on his account, a difference between ***Gen*** and its overt counterparts must be stipulated to capture the data in (68)–(69). If instead there are no cumulative Bona Fide Generic LFs, as on my account, the unavailability of cumulativity with overt Q-adverbs follows directly.

The second issue concerns bare plural vs. singular indefinite generics. Kirkpatrick argues both involve the same ***Gen***, but singular indefinites lack cumulative readings because they don’t introduce pluralities for ***Gen*** to range over. This predicts that if a sentence with a bare plural and conjunctive predicate is cumulatively true, then the corresponding singular indefinite with a *disjunctive* predicate should also be true.

(81)
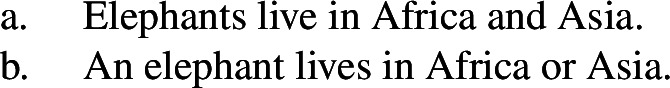

This is because the truth conditions in (81) follow from those in (79).

(82)
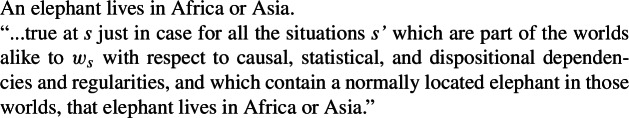

However, this is not correct, as is clear from the sentences in (83). Cumulative predication with kind-denoting plurals is compatible with accidentally-flavored generalizations, unlike corresponding disjunctive predicates with singular indefinite generics.

(83)

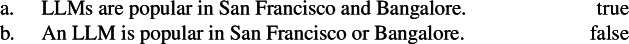

(84)
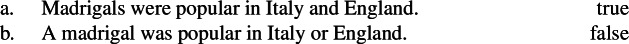

On my account, this is expected: in (84a), ‘madrigals’ can be interpreted outside the restriction of ***Gen***, yielding a true cumulative kind predication. In (84b), ‘a madrigal’ is interpreted inside the restriction, falsely implying a law-like connection between being a madrigal and being popular in Italy or England.

## The near-universal/existential alternation of English bare plurals in episodic sentences

This section is devoted to capturing bare plurals in episodic sentences. This problem can be decomposed into three points: (i) bare plurals can be read universally in episodic sentences, (ii) they can be read existentially in episodic sentences, and (iii) they display special scopal behavior. Section 5.1 is dedicated to (i), Sect. 5.2 to (ii), Sects. 5.3 and 5.4 articulate (i) and (ii) in the Romance-Germanic comparison, and Sect. 5.5 is concerned with (iii).

### A straightforward extension of the analysis to universal readings of bare plurals in episodic sentences

Unlike singular indefinites, bare plurals can be read near-universally in contexts where no item (e.g., habitual aspect) provides the sentence with generic force, specifically with stage-level predicates (Condoravdi 1994) and with verbs with episodic aspect (Dayal 2013; Chierchia 2022).^27^

(84)

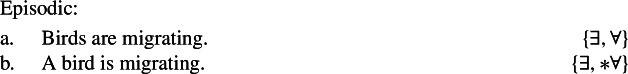

(85)
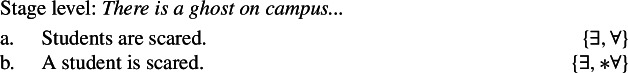

We can straightforwardly capture the near-universal readings of (84a) and (85a) by appealing, again, to an LF where the kind is interpreted with respect to the evaluation world and the predicate is distributed over it.

(86)

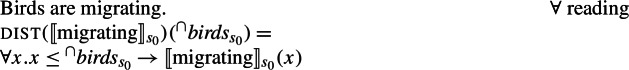

(87)
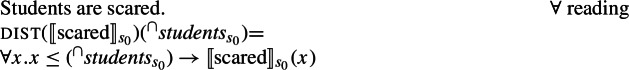

These uses of bare plurals are homogeneous and can be non-maximal:

(88)
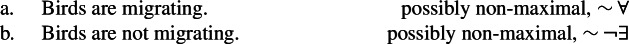

As noted in the introduction, the absence of QVEs is a strong argument against generic quantification in such sentences (Condoravdi 1994). A further correct prediction is that, given the discussion of homogeneity removal in Sect. 3.3, the lack of QVEs—and thus of ***Gen***—predicts removal with ‘all’ but not ‘always’. This is confirmed by (89)–(90).

(89)

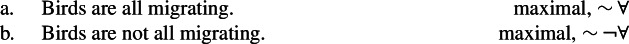

(90)
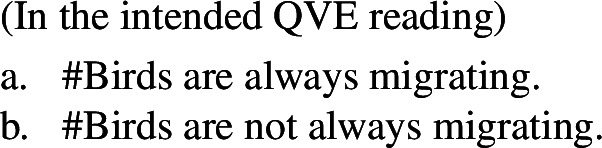

We also observe cumulativity in such non-generic environments, which are correctly predicted by straightforwardly extending the cumulative kind predication analysis argued for in Sect. 4, as shown in (91)—while unsurprisingly no such readings are available for the singular indefinite.

(91)
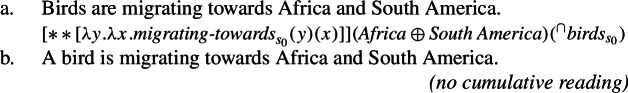

This further demonstrates the need for a cumulative kind predication LFs in which the kind is not interpreted in the restriction of a generic quantifier (notice similarly that (91)does not display QVEs).

(92)
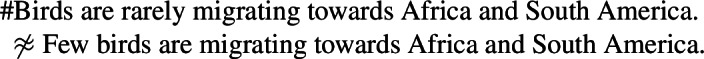

This thus adds to the point made in the previous section in favor of the idea that ***Gen*** can at the very least not be the only source of cumulativity in sentences like ‘Elephants live in Africa and Asia’, contra Kirkpatrick’s (2022) view.

### Existential episodic bare plurals

We have captured quasi-universal readings of episodic bare plurals via Distributive Kind Predication. At this point, the question arises concerning how this is to be pieced together with the account of sentences like (93), which at least on a descriptive level have existential force.

(93)

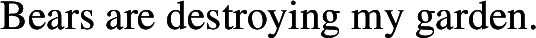

#### Two theoretical options

There are in principle two ways to conceive of such (descriptively) existential uses. **(i)** The first consists in saying that they are underlyingly existential; **(ii)** the second involves assuming that they constitute cases of extreme non-maximality.

**(i)**: **Existential bare plurals are underlyingly existential**

Specifically, the first line amounts to assuming that descriptively existential bare plurals receive a low-scoped existential interpretation, i.e., (93)receives a (low-scoped) version of the interpretation of (94).^28^

(94)

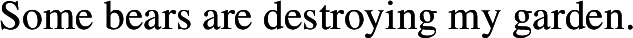

This line is taken by a quite diverse set of accounts. Chierchia (1998) argues that English BPs unambiguously denote kinds. When interpreted existentially, as in (95), it is argued that they do so as a result of the application of the last resort type-shifting operation known as Derived Kind Predication (DKP).

(95)

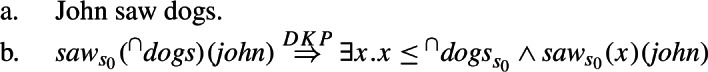

Longobardi (2001) instead sees English BPs as systematically ambiguous between a kind interpretation and a “weak indefinite” interpretation, responsible for (among other things) existential readings. In between Chierchia and Longobardi’s accounts, there are approaches that combine ambiguity and type-shifting, such as Cohen (2007, 2020).

**(ii)**: **Existential bare plurals are extreme cases of non-maximality**

The second line consists in saying that descriptively existential readings are extreme cases of non-maximality/exception tolerance. Extreme cases of non-maximality are known to be possible with definite plurals, as in, e.g., (96):

(96)
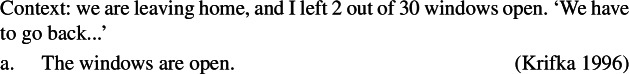

This line is generally taken by accounts that are concerned with the fact that bare plurals can have universal force, too, in episodic sentences and with stage-level predicates. Dayal (2013) was the first to argue for a revision of DKP that simply turns a kind into its maximal sum in a given situation.

(97)
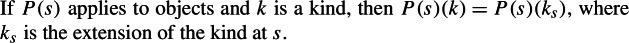

This rule has been used to explain both (descriptively) existential and near-universal readings of English bare plurals. Existential readings are seen as non-maximal interpretations, parallel to referential plurals (Lasersohn 1999, a.o.). In a similar spirit but in a different framework, Chierchia (2022) argues that the mechanisms behind homogeneity and non-maximality in definite plurals also underlie bare plurals. Here, verb thematic roles introduce discourse referents, which gain universal force via innocent inclusion of subdomain alternatives, yielding universal readings like in (5). Chierchia speculates that existential readings may result from the open-ended nature of the domain (2022, p. 506).

The insight presented here is compatible with both theoretical options, but there is reason to favor the view that existential readings arise from a distinct, underlyingly existential interpretation of bare plurals. In what follows, I present arguments for this view, and integrate them into the account of kind-denoting plurals developed in this paper. Specifically, I argue that existential interpretations stem from LFs where bare plurals are interpreted as *properties* (see Cohen 2020 for a related but non-identical view). Near-universal readings, by contrast, involve kind-denoting plurals over which the predicate distributes, as argued earlier.

#### Why existential bare plurals are not non-maximal

In what follows, I present an argument based on the interaction between bare plurals and epistemic adjectives like ‘unknown’ and ‘unidentified’.^29^ Such adjectives give rise to two distinct readings: a local reading where the adjective modifies the noun, and a nonlocal reading where it contributes propositional content (Abusch and Rooth 1997; Schwarz 2006, 2020; Morzycki 2016, 2020).

(98)
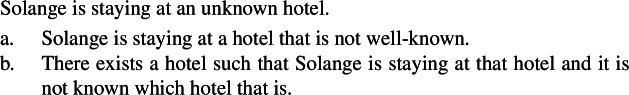

Consider the previously unnoticed contrasts in (99a)-(99b) and (100)[a]-(100)[b], which show that respectively ‘unknown’ and ‘unidentified’, if interpreted non-locally, block the universal reading of bare plurals.

(99)

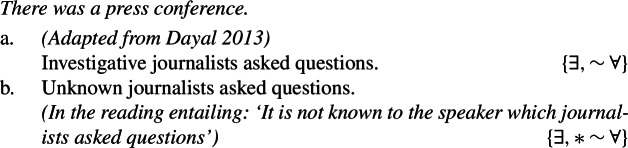

(100)
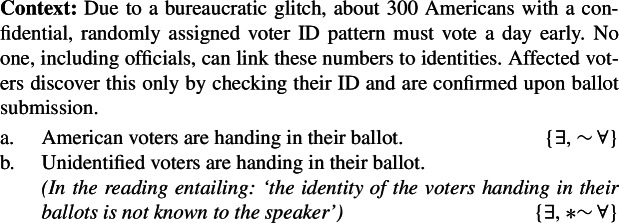

That (100a), but not (100b), allows for a universal reading is clearly shown by the contrast in felicity between the dialogues in (101a) and (101b).

(101)
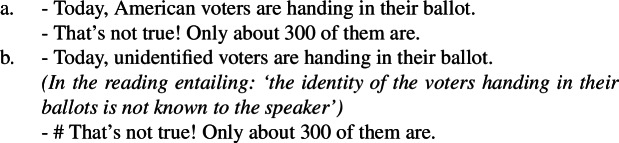

The unavailability of a near-universal interpretation in such episodic sentences correlates with the availability of kind predication.^30^ ‘American voters’, but not ‘unidentified voters’, supports kind predication. The same goes for ‘investigative/unknown journalists’.

(102)

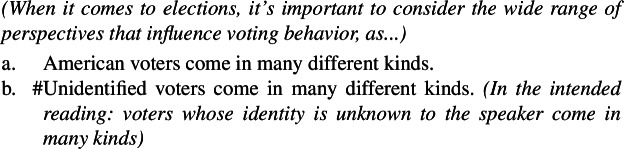

(103)
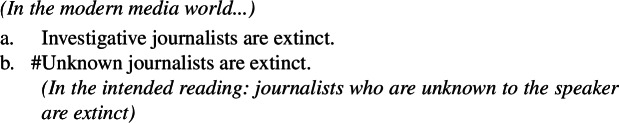

Approach **(i)** explains this data quite naturally, assuming there can be Distributive Kind Predication (and thus a near-universal reading) just in case a bare plural can denote a kind. For instance, the bare plural in (100a), but not in (100b), can receive a near-universal reading, since the former, but not the latter, can denote a kind, as shown by (102).

On **(ii)**-approaches (Dayal 2013; Chierchia 2022), the explanation is less natural. Existential bare plurals are analyzed as universal quantifications interpreted non-maximally, with context modulating strength. This makes a specific prediction: any bare plural that is existential in some contexts should admit a maximal construal in others. Yet bare plurals with epistemic adjectives, under their nonlocal reading, show a systematically weak interpretation across contexts, despite being structurally universal on **(ii)** views. Compare ‘philosophy professors’, whose non-maximality varies with context, to ‘unidentified professors’.^31^

(104)
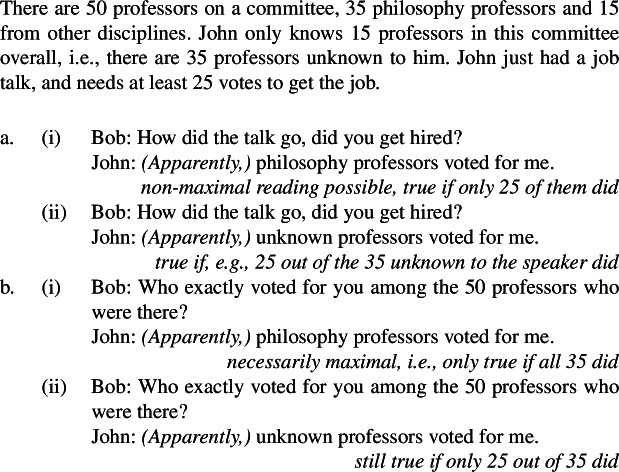

This is unexpected on a pragmatic account of non-maximality: if non-maximal construals are context-dependent, then the absence of context-dependency of ‘unknown professors’ in these cases is puzzling.

### Accounting for the ambiguity

I propose to account for the ambiguity displayed by English bare plurals in episodic sentences by taking them to be systematically ambiguous between (a) a kind-denoting LF and (b) a different LF, giving rise to their existential interpretation. Here I will argue that (b) is an LF in which the bare plural is interpreted as a property. Its existential force is given by a type-shifting operation parallel to DKP, which we will call Derived Property Predication (DPP).

(105)
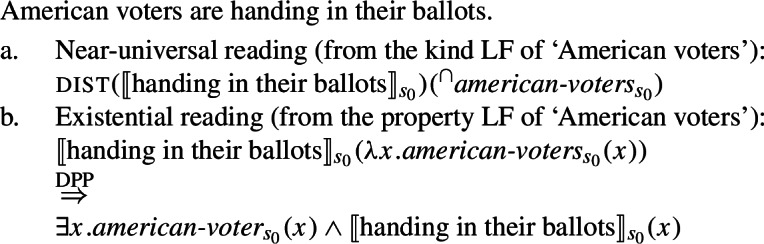

Since ‘unidentified voters’ cannot denote a kind due to its descriptive content (as shown by (102)), only the LF giving rise to the existential interpretation is left.

I take Carlson’s classic example in (93), ‘Bears are destroying my garden’, to be structurally ambiguous in an entirely parallel way. In this example, the existential reading is simply much more accessible, since the Distributive Kind Predication LF carries the very unlikely meaning that all existing bears happen to be destroying my garden right now. But crucially, this reading becomes salient in the right context, as in (106).

(106)
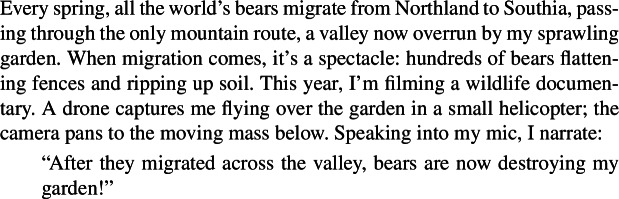

We have considered an argument against **(ii)**-approach theories in the previous section. At this point, some remarks on specific **(i)**-approach theories are in order, i.e., Chierchia (1998) and Longobardi (2001). In Chierchia’s (1998) account, English bare plurals always denote kinds, with existential readings derived via Chierchia’s DKP (see (95)). This makes the ‘unidentified voters’ case problematic: this expression does not support kind predication, yet receives a low-scoped existential reading. Moreover, since DKP applies to kinds and is a last-resort mechanism, we expect no ambiguity: either the kind fits the predicate, yielding a near-universal reading, or DKP applies, but kind predication is blocked. Thus, taking bare plurals as unambiguously kind-denoting undergenerates readings, unless predicates are themselves taken to be ambiguous between kind-level and individual-level construals.

Longobardi (2001), by contrast, predicts a full independence between the two readings. I will not adopt, here, Longobardi’s terminology describing existential bare plurals as “weak indefinites”, since it does not align with bare plural behavior under durative adverbs (see Sect. 5.5), which diverges from indefinites.

But the key point here is that in Longobardi’s, but not in Chierchia’s (1998) framework, existential readings of bare plurals arise from a different LF than the kind-level LF responsible for near-universal readings in episodic contexts. On this score, the present proposal is close to Longobardi’s.

### Italian bare and definite plurals as disambiguations of the uses of the English bare plural

A further point on which Chierchia (1998) and Longobardi (2001) diverge is the treatment of Italian bare plurals: Chierchia (1998) treats them as unambiguously kind-denoting, and thus takes them to give rise to existential interpretations via DKP, just like their English counterparts. Longobardi, instead, hypothesizes that because of a parameter-setting different from English, Italian bare plurals can only have a “weak indefinite” use. Translated into the present framework, Longobardi’s claim amounts to saying that Italian bare plurals can be interpreted only as properties.

Consider (107), showing that while English bare plurals can be ambiguous between a kind reading and an existential reading, Italian bare plurals instead only receive an existential reading. This constitutes a strong argument to think that Italian bare plurals and English bare plurals do not have the same interpretation.^32^

(107)

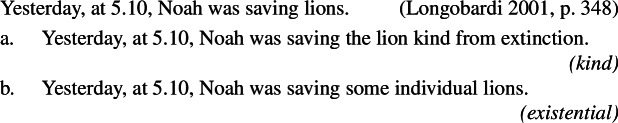

(108)
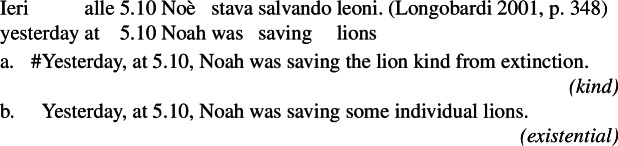

In this sense, we can see (99a) (‘investigative journalists asked questions’) as a counterpart of (107), with the only difference that in the LF of (99a) in which the bare plural is kind-denoting, there is *distributive* kind predication (while in (107b), presumably, simple kind predication).

Formulating examples parallel to (99a) in Italian with a bare plural subject, we only get an existential reading.

(109)

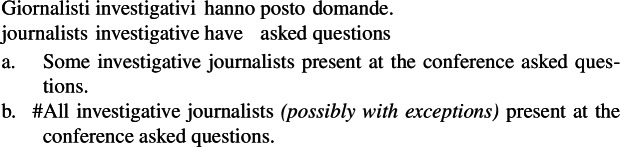

The Italian kind-denoting definite plural, instead, only has the near-universal reading, since it has a kind-denoting use, but not a property use. Sentences embedding it can only have an LF parallel to the Distributive Kind Predication LF of the English bare plural.

(110)
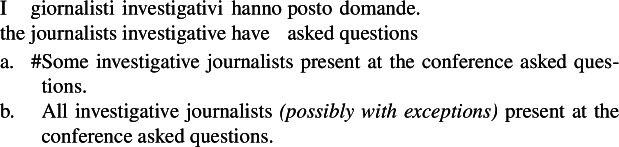

We can summarize the view suggested by this pattern as in (111)and (112).^33^

(111)

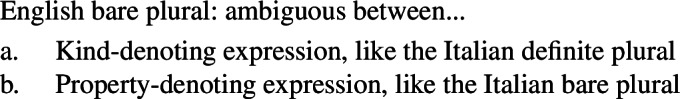

(112)
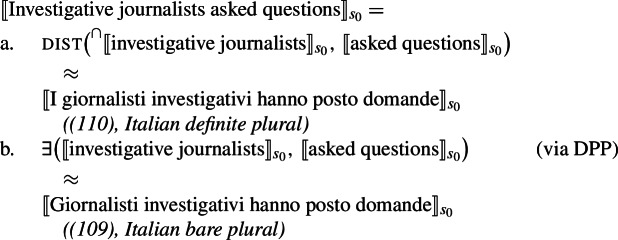

Additionally, since Italian bare plurals denote properties, we expect generic readings (like for singular indefinites in English and Italian). Because they cannot denote kinds, they should not yield non-generic accidental generalizations via Distributive Kind Predication (Sect. 3). Hence we expect the following pattern: the Italian definite plural allows law-like and accidental generalizations (see (113)), whereas the Italian bare plural allows only law-like ones (see (114); see also Cohen 2020 for similar ideas). This is what we find:

(113)

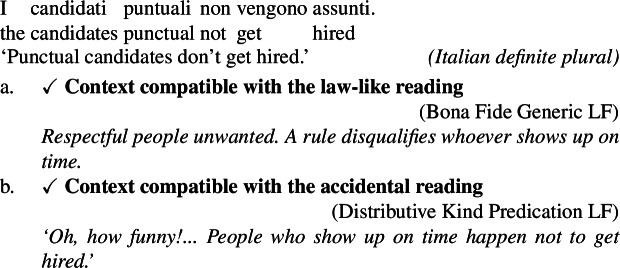

(114)

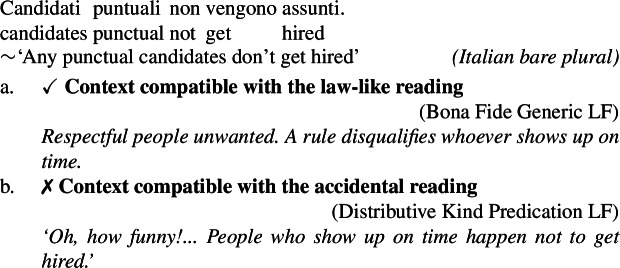

### Differentiated scope

Consider (115). While plain indefinites obligatorily receive high scope under durative modifiers, bare plurals are obligatorily low-scoped (see Chierchia 2022, 2023, a.o.).

(115)
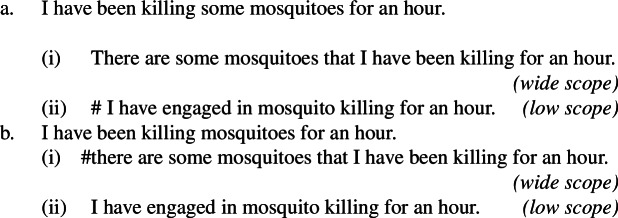

In this paper I hypothesized that all existential readings of bare plurals, both in English and in Italian, stem from a property LF. In this subsection, I show that this hypothesis is compatible with the facts in (115).

Chierchia (2023) explains the unavailability of the narrow-scope reading (115a-ii) via a scope economy constraint along the lines of the one proposed by Bassa Vanrell (2017): in a nutshell, with durative modifiers, scope-shifting operations are not allowed if they produce logically weaker interpretations. Since the low-scope reading of (115a) is strictly weaker than its wide-scope reading, it is disallowed.

Bare plurals, instead, receive obligatory low scope, as shown by (115b). Chierchia (2023) (see also Chierchia 2022) explains this by assuming that (i) English bare plurals unambiguously denote kinds, (ii) kinds can bear thematic roles, and (iii) the existential appearing below the durative modifier arises from (a form of) DKP—which applies locally. Because of this correct prediction, obligatory low scope of English bare plurals under durative modifiers has often taken to be evidence that these unambiguously denote kinds (see also Carlson 1977 for a first discussion of this data).

However, as Chierchia (2023) shows, any framework capturing the scopal behavior of sentences with durative modifiers needs to make space for an operation parallel to DKP allowing a *property*-denoting expression to take obligatory low scope below a durative modifier—which is exactly the DPP hypothesized in the present account. The main argument for this comes from French. French ‘des’ pseudo-partitives are not kind-denoting by any means, as confirmed by (116a), and yet they can take scope below durative adverbials and the like, as confirmed by (116b) (Chierchia 2023).

(116)
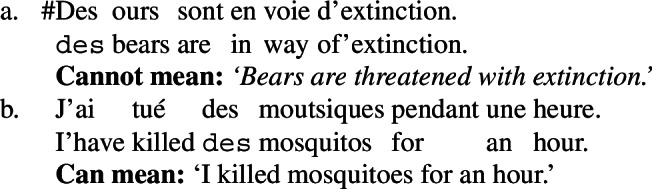

If we take traditional tests for kind-denotation like felicity with ‘extinct’ seriously (116a), then ‘des ours’ cannot denote a kind. As a consequence, Chierchia argues that (i) ‘des’ pseudo-partitives are property-denoting, (ii) properties can bear thematic roles, and (iii) some operation applying to properties needs to give us a low-scoped existential below the durative modifier (see also Chung and Ladusaw 2003; Dayal 2011; Gonzalez and Mihoc 2017 for related ideas).

Chierchia’s treatment of low-scope existential readings below durative adverbs in French is therefore compatible with the account proposed in this paper.^34^ On the present view, however, this treatment should in fact be extended to English and Italian bare plurals, since their existential readings are taken to stem from property-level LFs—DPP, proposed in Sect. 5.2, applies locally just like DKP. The possibility of such a mechanism specifically for Italian bare plurals is indeed recognized by Chierchia (2023, p. 82) too. This explains why Italian bare plurals behave like English bare plurals and French pseudo-partitives (as in (117)), and why Italian definite plurals lack such readings (as in (118)), since they are unambiguously kind-denoting.

(117)

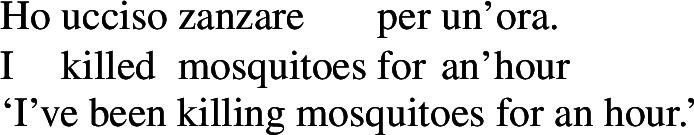

(118)
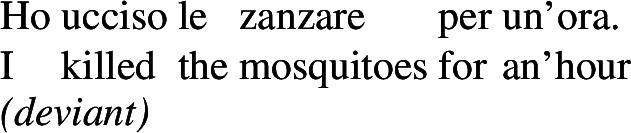

One last point that should be mentioned is that on the present approach, the felicity of English and Italian bare plurals in predicative position (i.e., as codas of copulas) follows straightforwardly, since they denote properties. On this score, English and Italian bare plurals are like French ‘des’ pseudo-partitives and unlike Italian kind-denoting definite plurals.

(119)
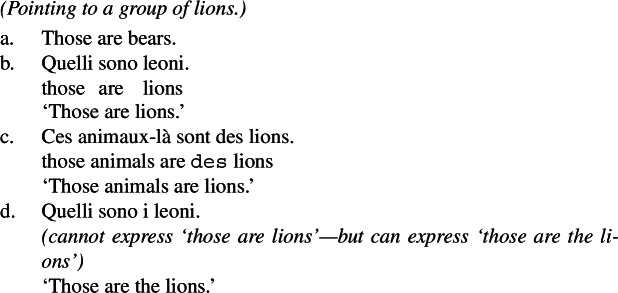

Before moving on, let me discuss some ideas put forward within the Chierchia-Dayal Neo-Carlsonian tradition concerning the bare plural ‘parts of this machine’. This bare plural arguably cannot denote a kind, as shown in (120), and can take wide scope over negation.

(120)

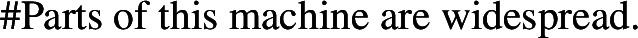

(121)

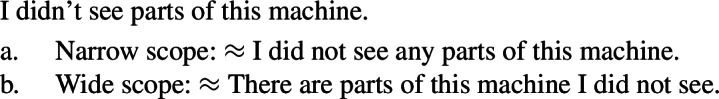

(122)
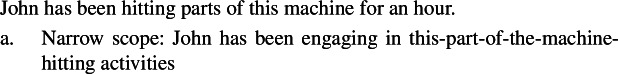

In particular, it is assumed in this tradition that when a bare plural cannot denote a kind because of its descriptive content, it cannot be type-shifted by ^∩^, and is thus type-shifted by ∃, the type-shifting operation just below ^∩^ in the hierarchy hypothesized for type-shifters in this tradition. Crucially, this process is entirely different from DKP: DKP kicks in when there is a mismatch because a predicate of individuals is applied to an argument that denotes a kind, while ∃ type-shifts a bare argument when for some reason it cannot denote a kind. The crucial difference is that DKP is predicted to occur at the lowest possible scope site, while ∃ can take wide scope, since it practically turns the expression into an indefinite. Thus, Chierchia and Dayal argue that they can predict the possibility of wide scope of ‘parts of this machine’.

Notice however that the same does not hold of the kind-incompatible expressions we have discussed in Sect. 5.2.2 like ‘unidentified voters’, which behave scopelessly under negation, just like a vanilla bare plural like ‘American voters’.

(123)

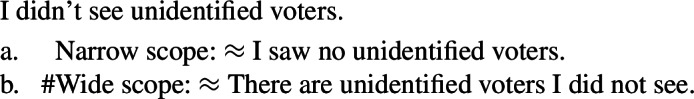

(124)
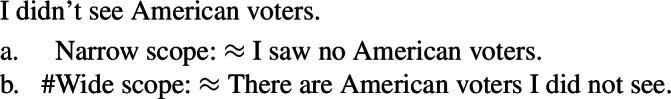

This parallel behavior suggests that the mechanisms giving rise to the existential reading of ‘American voters’ and to the (only) existential reading of ‘unidentified voters’ are the same.

This also suggests that the scopal behavior of ‘parts of this machine’ may be due to ‘parts’, and not a general fact. To this point, it should be mentioned that Krifka (2003) argues that if there is a wide-scope reading of (121), it might be because of peculiarities of ‘parts of’ that allow it to act as a quantifier, and not because ‘parts of this machine’ cannot denote a kind. Incidentally, Krifka also mentions that the judgments on ‘parts of this machine’ are not as clear as they have sometimes made to be.^35^

Thus, it could be that in ‘parts of this machine’, ‘parts’ may be interpreted either as a bare noun or as a quantifier. In its bare noun interpretation, it cannot denote a kind; but just like in the case of epistemic adjectives, the property-level reading remains, yielding a low-scope existential reading. In the quantifier interpretation of ‘parts’, instead, ‘parts of this machine’ can QR above negation.

An independent reason to think that ‘parts’ can function as an existential quantifier (perhaps due to its role in partitive constructions) comes from Italian. Italian bare plurals cannot occur as subjects unless modified, a fact often attributed to licensing constraints on the null D heading them (Chierchia 1998, a.o.). Yet *parti* ‘parts’ can appear unmodified in subject position and take wide scope, even without indexical modification (in Dayal’s sense, e.g., ‘parts of this machine’). It could be that *parti* is interpreted as a quantifier in D, supporting the view that English *parts* likewise might function as a partitive quantifier.

(125)

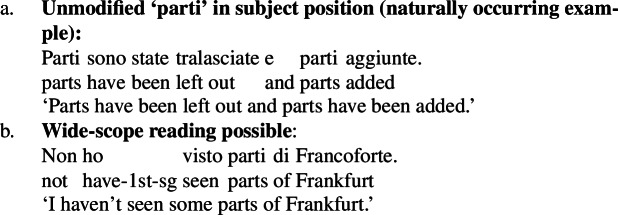

## Discussion and open issues: singular and plural definites

### English bare and definite plurals are not interchangeable

A natural question to ask, at this point, is why English bare and definite plurals are not interchangeable, since on this view, when the world variable of kinds is saturated their LF is equivalent to the LF of definite plurals.^36^ In fact, **(i)** English bare plurals cannot be used in some environments where definite plurals can be used, as shown by (127) for episodics and (129) for individual-level predicates; **(ii)** English definite plurals cannot be used in some environments where bare plurals can be used, as shown by (126) for episodics and (128) for individual-level predicates.^37^

(126)

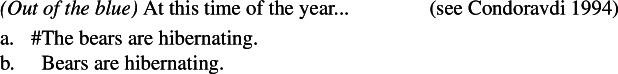

(127)

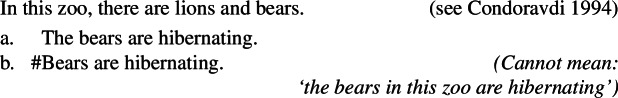

(128)

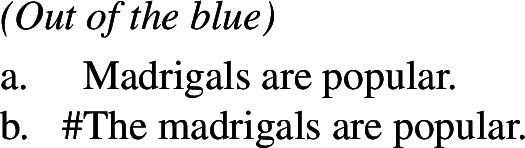

(129)
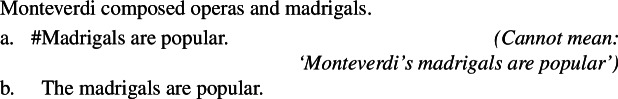

Explaining (i) is straightforward. Referring back to the specific set of bears or madrigals introduced in the context requires a domain restriction variable; such variables are known to be tied to the presence of an overt determiner (von Fintel 1994; Feinmann 2022), which the bare plural lacks.

(130)
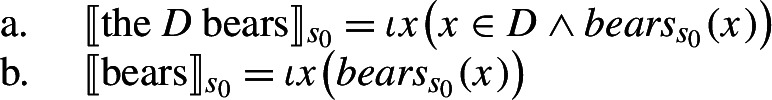

Point (ii) is a known problem that concerns definites, rather than bare plurals, first noticed by Heim (2011), who pointed out that English definite plurals cannot be used in the absence of a non-trivial domain restriction. Her original examples make use of collective, non-kind-specific predicates, but they are completely parallel to the ones above.

(131)
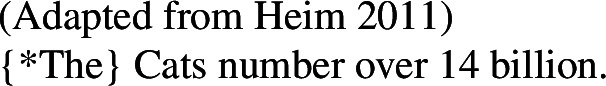

In Heim’s words, “even if we choose predicates that elsewhere have no difficulty applying to pluralities of ordinary individuals [*rather than kind predicates like ‘extinct’*], we still don’t get good sentences when the definite plural is not contextually restricted and is intended to pick out the sum of all existing instances of the noun”. In other words, since Carlson (1977), the infelicity of English definite plurals with predicates like ‘extinct’ has been attributed to their inability to denote a kind; but sentences like (131)(and (126) and (128)), as Heim notes, do not contain kind predicates in any relevant way, nor are they generic.

A link between domain restriction on one side and genericity and kind predication on the other has been observed by Guerrini and Spector (2025), who illustrate the generalization in (132).

(132)
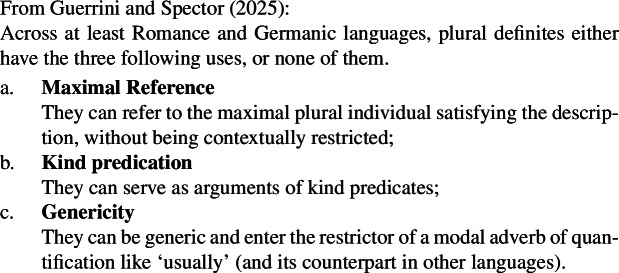

This generalization poses at least two so far unresolved questions for theories of kind predication and definiteness: (i) why can kinds not be created out of domain-restricted definites? (for instance, in Chierchia’s 1998 framework, this would be via abstraction of their world variable); and (ii) why are English definites incompatible with trivial (i.e., maximal) domain restrictions? Concerning (ii), one may speculate that the definite plural is incompatible with maximal domains due to competition with the bare plural, which due to the lack of domain restriction variables is only compatible with the unrestricted domain provided by discourse.

These questions remain open. All the cases of Distributive Kind Predication we have considered in this work are in fact instances of what Guerrini and Spector call Maximal Reference. Following this discussion, the main claim of the paper can be recast as stating that bare plurals have definite uses that explain a great deal of their distribution. Of course, given this, one may prima facie expect them to have the same distribution as definite plurals. However, the above discussion shows that the distributional differences between the two expressions are not due to bare plurals not having the definite uses hypothesized by this paper. Rather, they can be seen as stemming from them having different constraints on domain restriction as compared to definite plurals.

### Singular kind-denoting terms strikingly diverge from plural kind-denoting terms

So far, I have left kind-denoting singular definites undiscussed in this paper.^38^ It is well-known that singular definites support generic quantification, and are compatible with law-like, but not accidental, readings (see Krifka 1995, as well as Dayal 2004b, p. 433).

(133)
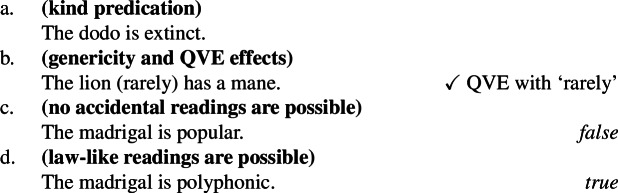

A fuller account of singular kind terms must be left to future work, but the present framework can in fact offer an explanation of it, for the following reasons. Singular kinds are generally assumed to have a different LF from plural kinds (Chierchia 1998; Dayal 2004b). Dayal, for instance, likens them to group nouns like *committee*, in contrast to plural kinds, which correspond to *(the) members of the committee*. Group nouns are often treated as atomic (Barker 1992; Schwarzschild 1996). Following de Vries (2015, 2017), I assume that distributive readings with such nouns do not involve a syntactic operator like dist, but arise via “context-based reasoning about parts and wholes in relation to a predicate” (de Vries 2017, p. 195).

(134)
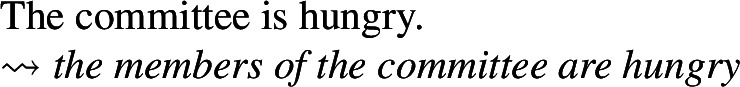

Given Dayal’s analogy between singular kinds and group nouns and de Vries’s treatment of distributive readings with group nouns, the absence of accidental readings is expected on the present account. I indeed derive accidental readings from the presence of an explicitly represented dist, which in the Distributive Kind Predication LF keeps the bare plural from restricting ***Gen***. If such an operator is not present at LF, accidental readings are predicted not to arise.

The possibility of a law-like reading is instead expected: in the present account, this reading is due to ***Gen***, and adverbial generic quantification is clearly possible with singular definites given the possibility of QVEs illustrated in (133b) (see also Chierchia 1998, p. 381, a.o.).

Finally, the lack of an explicitly represented distributive operator would also explain why episodic readings are much more restricted with singular kind terms than with plural kind terms, as shown by (135a,b) (see Chierchia 1998, p. 379). And taking cumulative kind predication to arise from the explicitly represented ∗∗-operator, as I argued, we also correctly predict the unavailability of the cumulative reading of (136b).

(135)

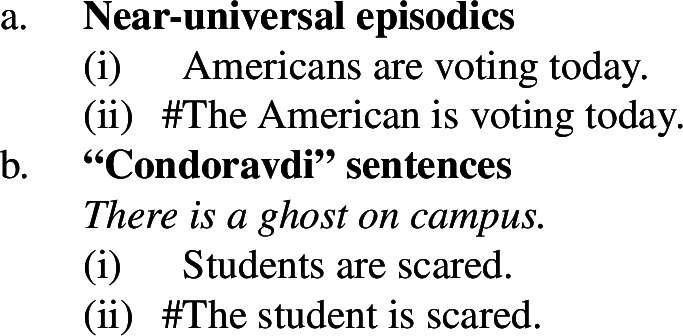

(136)

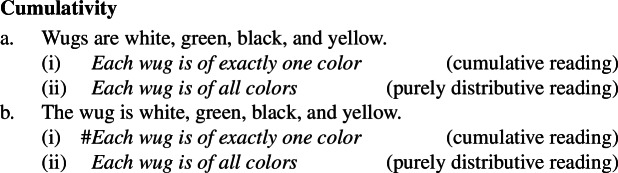

## Conclusion

Let us summarize what we have done. Some plural terms can both denote kinds and occur in sentences expressing generalizations. In such sentences, they have a very different distribution from singular indefinites. By assuming that plural kind-denoting terms can denote the maximal sum of kind members in the evaluation world, and by applying distributive and cumulative operators to these sums, we have seen that we can fully predict the distinct distribution patterns displayed by kind-denoting plurals and singular indefinites.

More specifically, we’ve demonstrated that some sentences traditionally assumed to contain generic quantification at LF, as for instance accidental generalizations, in fact do not.

(137)

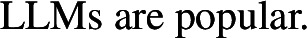

This allowed us to capture the pattern that unites accidental generalizations like (137)and near-universal construals of episodic sentences such as (138): both are supported by kind-denoting plurals, but not by singular indefinites.

(138)

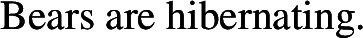

Generic quantification can indeed simply not be the source of the near-universal construal of (138), since sentences like (138)do not display QVEs, unlike uncontroversially generic sentences.

(139)

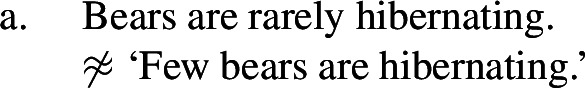

(140)
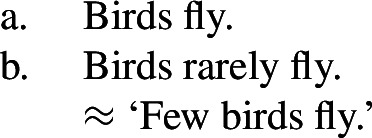

The fact that (137), in its salient accidental reading, in fact does not involve generic quantification is not without consequences. Traditionally, all generalizations involving kind-denoting plurals have been thought to uniformly involve ***Gen***. For this reason, the distinct distributions of English bare plurals and singular indefinites have often been taken as an argument to complexify the interpretation of the generic quantifier, as for instance in Greenberg’s (2007) theory of the exception tolerance of generics. Recognizing that many of such sentences have a reading in which in fact there is no generic quantification over members of the kind makes the task of understanding their exception tolerance more manageable.

The fact that (137), in its accidental reading, does not involve generic quantification has consequences. Generalizations with kind-denoting plurals were long thought to uniformly involve ***Gen***, leading to increasingly complex accounts of its meaning. But recognizing that many such sentences lack generic quantification makes the issue more tractable.

For instance, we no longer need to complicate the interpretation of ***Gen*** to explain why bare plurals and singular indefinites differ in exception tolerance. Non-generic generalizations with kind-denoting plurals can be treated like referential plurals (see also Mari 2010), since their exception tolerance can be removed via ‘all’. For genuine generics, the comparison is with singular indefinites. Generic quantification itself displays homogeneity and non-maximality, removable via ‘always’. This explains why, in sentences expressing generalizations, ‘all’ removes homogeneity for referential and kind-denoting plurals, but not singular indefinites; while ‘always’ removes it for kind-denoting plurals and singular indefinites, but not for referential plurals.

(141)

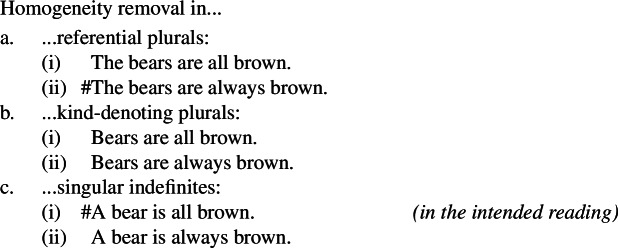

We said we expect Distributive Kind Predication to display a kind of non-maximality similar to referential plurals. In this connection, while providing a Distributive Kind Predication analysis for sentences like (140), we demonstrated that English existential episodic bare plurals like (142)clearly have a distinct interpretational source from kind predication, and thus do not constitute undocumented and extreme cases of non-maximality.

(142)

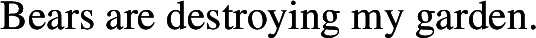

This is essentially because expressions that do not admit a kind-level interpretation nor a near-universal interpretation in episodic sentences still admit an existential interpretation in episodic sentences:

(143)

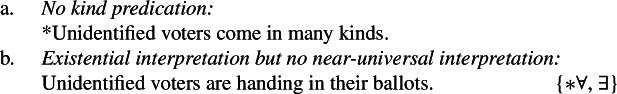

This allowed us to see that English bare plurals are in fact ambiguous between a kind-level and a property-level interpretation, as has already been advocated on different grounds (see Cohen 2007, 2020, as well as Longobardi 2001 for a very similar idea). Moreover, we saw that their two different uses find exact and unambiguous counterparts in Italian, where the bare plural can only be existential in episodic sentences, while the definite plural can only be universal, as re-illustrated in (145).

(144)
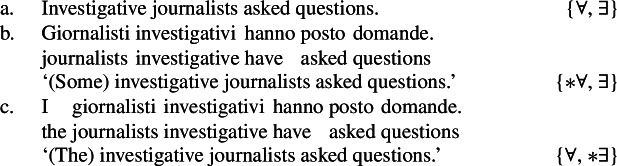

This can be made sense of within a framework in which Italian bare plurals unambiguously denote properties, Italian definite plurals unambiguously denote kinds, and English bare plurals are ambiguous between kinds and properties, as re-summarized in the diagram in (145):

(145) .

Let me conclude, then, with a perspective for future research. As we have seen, the data in (143) and (144) is hard to accommodate within Chierchia’s (1998) theory of cross-linguistic variation of the interpretation of bare nouns. I believe, however, that it suggests an overall perspective on these phenomena that is much in line with its original spirit. Chierchia (1998) proposes that there is a parameter regimenting the mapping of items that are syntactically labeled ‘N’ onto semantic objects, specifying whether they can map onto an argument (i.e., a kind) and/or onto a predicate (a property). Chierchia proposes that English can map nouns both onto kinds and onto properties, while Italian can map them only onto properties.

(146)
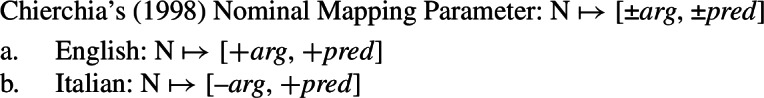

If we understand (146a) as stating that English bare plurals can *systematically* denote either semantic object (departing on this aspect from Chierchia 1998), we obtain their pervasive ambiguity that is summarized in the diagram in (145). Just like Chierchia (1998), we still expect that the only way Italian has to achieve reference to kinds is through a definite determiner. As to the Italian bare plural, the syntactic literature abounds with evidence that it is headed by a null determiner D^0^ (see Chierchia 1998; Longobardi 2001, and references therein). There has been much debate on what the semantics of this determiner is; within the framework just sketched, there is no reason to not treat it as simply semantically vacuous, passing onto the next node the property input it takes. This has the additional advantage of correctly predicting that exactly those expressions giving rise to low-scope existential readings are also grammatical as codas of copulas.

There is still much to be done to understand how this framework could be extended beyond Germanic and Romance, so this work is an attempt-in-progress. I hope however to have demonstrated the fruitfulness of two points: (i) hypothesizing that forms of kind predication obtain outside the boundaries traditionally attributed to it, both for our understanding of genericity and of non-generic generalizations, and (ii) taking them to be essentially parallel to referential plural predication in terms of semantic composition (e.g., distributivity) and pragmatic behavior (e.g., homogeneity and non-maximality).

## Footnotes

1 A less developed version of this claim appeared in Guerrini (2024).

2 Examples (3) are about Large Language Models. This is a switch from the classic example ‘Madrigals are popular/polyphonic’/‘A madrigal is popular/polyphonic’. I made the switch mostly because many people I consulted for judgments did not know what madrigals are; but all the theoretical claims I make in this paper apply to classical examples of the ‘flavors of genericity’ puzzle just as well.

3 A notational point: I typographically distinguish the generic silent quantificational adverb from the modal quantifier that its lexical meaning embeds by writing the former in boldface LF and the latter in small caps:

- Quantificational adverb:
  ***Gen***
- Modal quantifier:
  gen

That means:

4 As noted by a reviewer, while this is the standard view of (16a), familiarity/novelty conditions apply to kinds much like to individuals. As Geurts (2001) and Dayal (2004b) observe, singular indefinites can introduce new kinds (e.g., ‘Fred invented a new machine’ = ‘a new kind of machine’), whereas definite singulars require the kind to be already salient. Thus, ‘Fred invented the pumpkin crusher’ presupposes an established kind, unlike ‘Fred invented a pumpkin crusher’, which introduces one; see also Dayal (To appear). Thus, when I write that an expression cannot denote a kind, I mean that it cannot denote a *familiar* kind—I put novel kinds aside and leave the investigation of article choice to another occasion.

5 Not any quantificational determiner can enter the restriction of ***Gen***. The usual test to understand whether a GQ can enter the restrictor of ***Gen*** is to see whether it can provide a suitable restriction for overt Q-adverbs. This is the case of ‘two’:

However, this is not so for, e.g., ‘every’:

This issue is beyond the scope of the present paper, but since Q-adverbs can be restricted by a property, we do expect there to be an alignment between those quantifiers that can(not) receive property-level interpretations in, e.g., copulas and those that can(not) restrict ***Gen***, which seems to be the case.

The reasons why quantifiers like ‘every’ cannot receive property-level interpretations are discussed, e.g., in McNally (1998).

6 Note that here I am simplifying the denotation of VPs, for ease of exposition; but things are likely more complex. See Sect. 3.4.

7 In the literature on genericity, sentences like (21d) have been mostly described as infelicitous. They are arguably best described as false instead. This is because dialogues such as the following are completely natural according to the two native speakers of English that I consulted:

8 Florian Schwarz (p.c.) pointed out to me that (25a) is felicitous in contexts where ‘usually’ quantifies over entire worlds. Take a number of computer simulations of the evolution of species on Earth in different conditions: ‘lions usually go extinct’ is perfectly natural. He points out that this could be taken to suggest that ‘usually’ can be construed, in the right context, as ranging over whole world-scenarios as units. By contrast, the default construal of the kind targets sub-world events or situations, which would explain the tension in ordinary uses.

9 See, e.g., Liebesman (2011) for a fully non-quantificational theory of generics.

11 For simplicity, I here assume that dist can only distribute down to atoms.

12 The same could be achieved by adopting, instead of dist, Schwarzschild’s ∗ operator. As is standard, the ∗ operator would have to be always obligatorily inserted. In this case, the cover function in the interpretation of * would simply return atoms composing the kind.

13 Some assume that Italian definite plurals do not directly denote kinds, unlike English bare plurals, but do so via abstraction over the world parameter (e.g., Chierchia 1998). This is not crucial here: what matters is that in the Distributive Kind Predication reading, the subject DP is evaluated at the world of evaluation, while in the Bona Fide Generic reading, its world variable is bound by ***Gen***. On Chierchia’s view, this corresponds to $\iota x(\textit{lion}_{s_{0}}(x))$ vs. $\lambda s. \iota x(\textit{lion}_{s}(x))$, but these yield equivalent LFs to those shown above.

14 I find it more straightforward to capture existential readings of these indefinites choice-functionally, but the account doesn’t hinge on this; we could represent them as plain existentials just as well.

15 ***Gen*** can draw on different modal bases. It’s long been noted that sentences like (32)are most naturally read deontically (see Krifka et al. 1995).

Importantly, the corresponding bare plural generic is then ambiguous between a modal (here, deontic) and a descriptive reading, mirroring the structural ambiguity argued for in this paper, with the former from the Bona Fide Generic parse and the latter from the Distributive Kind Predication parse.

16 In (32) and henceforth, I use ‘∼’ as a symbol for approximation, i.e., ∼∀ as ‘approximately universal’.

17 That this cannot be due to the negation occurring within the scope of gen was shown by Magri (2012) through examples like (36): it is impossible to negate (35a) while maintaining that some or many lions hunt.

See also Križ (2015).

18 A treatment of homogeneity and non-maximality based on trivalence (like Križ’s) suggests a very natural correspondence between a trivalent entry for dist and a trivalent entry for ***Gen***, roughly as in (40)—which may incidentally help with the homogeneity and exception-tolerance of ***Gen***.

19 For this to work, we have to suppose that in these cases, ***Gen*** is not looking for an 〈*s*,*e*〉 object, but for an *e* object.

20 A less developed version of the argument from the Italian subjunctive appeared in Guerrini (2025).

21 An additional data point is worth mentioning. Greenberg (2012) points out that ‘happen to’ is only compatible with bare plurals, not with singular indefinites, and with bare plurals it has only an accidental reading.

She treats ‘happen to’ as a circumstantial modal fixed by facts about the subject in the actual world. In my framework, it can be argued that, anchored at the evaluation world, ‘happen to’ resists modal quantification by ***Gen***, blocking the Bona Fide Generic LF otherwise available. With bare plurals, a Distributive Kind Predication LF survives, yielding the accidental reading. In this sense ‘happen to’ is the mirror image of the Italian subjunctive, which forces law-likeness by blocking the Distributive Kind Predication LF. I thank Yael Greenberg and Manfred Krifka for encouraging me to discuss this.

22 And in this case, on a Chierchia-like theory, there will be ***Gen*** below ∗∗; on a Dobrovie-Sorin-like theory, there will be ***Hab*** below ** (see Sect. 3.4).Importantly, Quantifier Raising above ****** is independently needed to handle some cases of cumulativity with referential plurals (see Beck and Sauerland 2000; Chatain 2021).

23 Note that the possibility of a cumulativity operator under GEN plausibly implies the possibility of a DIST (or a *-operator) under GEN. In this case, GEN would range over sums (just like it does in (65)) and DIST (or *) would appear in the scope of GEN. I don’t discuss such an extension in this paper, as for simplicity I have restricted myself to cases in which GEN and DIST range over atoms. Stacking of GEN and DIST (or, for that matter, of the cumulative operator, as we will see) does not affect the main distinction between bona fide generics and distributive kind predication: what matters is what operator is highest and what appears in its restrictor.

24 And thus below habitual marking if we think that ***Gen*** comes from an AspP.

25 I confirmed these judgments (as well as those in (69)) with three native English speakers, unaware of my proposal and not trained in linguistics. Informants were shown two pictures and asked whether the sentence could describe the scene. The cumulative scenario depicted 12 creatures: three entirely black, three white, three green, and three red. The “simultaneous” scenario depicted 12 wugs, each striped black, white, green, and red.

26 Here is a naturally occurring example:

The distributive reading of (68a) is unlikely: it’s odd to say tigers are *both* white and orange but not black, given their well-known black stripes. Rather, (68a) suggests there are (mostly) orange and (mostly) white tigers.

27 One might ask whether (85) is similar to (84), since in (85) ‘students’ refers only to those on campus. This restriction likely arises from a global mechanism, i.e., evaluation relative to a subpart of the world or a situation (cf. Dayal 2013), rather than from a domain restriction variable on the determiner (see von Fintel 1994; Feinmann 2022). As Guerrini and Spector (2025) argue based on examples by Condoravdi, Condoravdi-type restrictions are global: in examples like (i), plural definites can be further restricted in ways bare plurals cannot.

As a reviewer notes, this kind of global restriction also appears in (ii), which refers only to lions in Africa.

28 For low scope, see Sect. 5.5.

29 For two other arguments against non-maximality-based views of existential readings of bare plurals, see Guerrini and Spector (2025).

30 A reviewer points out that in their local readings, these adjectives do support kind predication:

Crucially, under this reading the universal reading becomes available again:

31 I prompted three native speakers of English for these judgments. Importantly, notice that the definite plural ‘the philosophy professors’ behaves exactly like ‘philosophy professors’ in these examples.

32 For similar arguments that Spanish bare plurals can only denote properties, see McNally (2004).

33 A point worth noting concerns the (im)possibility of kind predication with Italian bare plurals. Judgments in the literature diverge: Chierchia (1998) finds them acceptable as arguments of kind predicates; Longobardi (2001) does not. I consulted five native Italian speakers—two linguists, three non-linguists, none aware of my account. All align with Longobardi:

If these judgments are correct, the present account predicts them. Chierchia (1998) notes that some contexts improve bare plural kind predication, e.g.:

In such cases, a last-resort type-shift may rescue the sentence by mapping the property to a kind. I leave this controversial issue to future work.

34 A reviewer notes that in Carlson’s original statement of the differentiated scope puzzle, sentences like (117)were counted as part of the problem:

I suggest (116)is direct kind predication. Were it individual-level it would wrongly imply that (some/each) individual wolf grows as I go north (Carlson 1977). Hence ‘keep getting bigger as I go north’ is true of a kind iff as one proceeds farther north its instances are progressively larger. Crucially, this is supported by the fact that such predicates also combine with definite plurals:

With durative adverbs, bare vs. definite plurals diverge: bare plurals take obligatory narrow scope; definites obligatory wide scope.

Hence (i) is not the same phenomenon as (115a).

35 It should be mentioned that Dayal (2013) argues that wide scope is only possible for a subset of such nouns, mentioning some other expressions that she takes to be simply ungrammatical. This is the case of ‘fibres of this rope’ and ‘stones of this monument’. However, I think that this judgment needs reexamination, for consider examples (125)-(126), judged perfectly natural by the three native speakers of English I consulted:

36 I thank two reviewers for encouraging me to discuss this.

37 This pattern is of course not visible in Italian since referential and kind-denoting plurals are both expressed with definite plurals.

38 I thank an anonymous reviewer for encouraging me to discuss this.
